# Backbone Interactions Between Transcriptional Activator ExsA and Anti-Activator ExsD Facilitate Regulation of the Type III Secretion System in *Pseudomonas aeruginosa*

**DOI:** 10.1038/s41598-020-66555-z

**Published:** 2020-06-18

**Authors:** Manisha Shrestha, Robert C. Bernhards, Yichen Fu, Kylie Ryan, Florian D. Schubot

**Affiliations:** 1 Department of Biological Sciences, Virginia Polytechnic Institute & State University, Washington Street, Blacksburg, VA 24060 USA; 2 Department of Radiation Oncology, UT Southwestern Medical Center, Dallas, TX 75390 USA; 3 Defense Threat Reduction Agency, Fort Belvoir, VA 22060 USA; 4 U.S. Army Combat Capabilities Development Command (CCDC) Chemical Biological Center, Aberdeen Proving Ground, MD 21010 USA; 5 College of International and Extended Studies, San Jose State University, San Jose, CA 95310 USA

**Keywords:** Microbiology, Molecular biology

## Abstract

The type III secretion system (T3SS) is a pivotal virulence mechanism of many Gram-negative bacteria. During infection, the syringe-like T3SS injects cytotoxic proteins directly into the eukaryotic host cell cytoplasm. In *Pseudomonas aeruginosa*, expression of the T3SS is regulated by a signaling cascade involving the proteins ExsA, ExsC, ExsD, and ExsE. The AraC-type transcription factor ExsA activates transcription of all T3SS-associated genes. Prior to host cell contact, ExsA is inhibited through direct binding of the anti-activator protein ExsD. Host cell contact triggers secretion of ExsE and sequestration of ExsD by ExsC to cause the release of ExsA. ExsA does not bind ExsD through the canonical ligand binding pocket of AraC-type proteins. Using site-directed mutagenesis and a specific *in vitro* transcription assay, we have now discovered that backbone interactions between the amino terminus of ExsD and the ExsA beta barrel constitute a pivotal part of the ExsD-ExsA interface. Follow-up bacterial two-hybrid experiments suggest additional contacts create an even larger protein–protein interface. The discovered role of the amino terminus of ExsD in ExsA binding explains how ExsC might relieve the ExsD-mediated inhibition of T3SS gene expression, because the same region of ExsD interacts with ExsC following host cell contact.

## Introduction

*Pseudomonas aeruginosa* is a ubiquitous Gram-negative bacterium that causes opportunistic infections in many organisms including humans^1,2^. *P*. *aeruginosa* accounts for an estimated 13% of all intensive care unit infections in the United States^3^. *P*. *aeruginosa*-associated pneumonia has a mortality rate of nearly 50%^4^, while chronic lung infections caused by *P*. *aeruginosa* are the leading cause of mortality among cystic fibrosis patients^5,6^. The type III secretion system (T3SS) has emerged as a key virulence determinant of acute infections^4^. This secretion apparatus acts like a complex molecular syringe to transport four well-characterized toxins, ExoS, ExoT, ExoU, and ExoY, directly from the cytosol of the bacterial cell into the eukaryotic host cytoplasm^7–9^. Their respective effects include disruption of the host cell actin cytoskeleton, inhibition of host cell cytokinesis, phospholipase-induced cell death, and inhibition of phagocytosis^7–9^. Transcription of the effector genes and the structural and regulatory components of the T3SS is controlled through a number of regulatory mechanisms^10–12^. A cascade involving the four proteins ExsA, ExsC, ExsD, and ExsE has been shown to mechanistically link host cell contact with upregulation of T3SS-related promoters by the transcriptional activator ExsA^13–21^. Prior to infection, ExsA is inhibited by ExsD through the formation of a 1:1 complex that prevents ExsA homo-dimerization and promoter binding, while the T3SS chaperone ExsC is sequestered by the 81 amino acid protein ExsE^17,19^. Upon host cell contact, ExsE is translocated into the host cell. Following secretion of ExsE, the liberated ExsC protein sequesters ExsD, which enables ExsA to bind to its cognate promoters and thus stimulate T3SS gene expression^13–19^. ExsA is a class II type transcriptional activator belonging to the prominent AraC/XylS family of proteins^20^. AraC proteins are involved in regulating a variety of processes, including cellular metabolism and virulence^22–31^. The characteristic AraC domain consists of two helix-turn-helix motifs that bind to cognate promoter elements^26^. Canonical AraC-type proteins feature an additional amino-terminal (N-terminal) dimerization and ligand-binding domain that mediates regulatory mechanisms through interactions with cellular signaling molecules^28,31–34^. ExsA, a 278 amino acid protein, is a prototypical AraC protein. The carboxy-terminal AraC domain interacts with promoter elements and the 4.2 region of σ^70^, whereas the N-terminal domain (NTD) facilitates dimerization and binds to the anti-activator ExsD^13,14,18,35^. There are ten known ExsA-dependent promoters. Activation of these promoters requires the successive binding and dimerization of two ExsA molecules near the −35 elements^20,35,36^. ExsD sequesters ExsA by forming a 1:1 complex prior to host cell contact^14^. This direct interaction was first demonstrated using a bacterial-two-hybrid assay^18^ before two further studies revealed that ExsD interferes with ExsA function by disrupting the ExsA dimer and preventing efficient promoter binding^14,37^. However, the exact mechanism by which ExsD achieves this remains unclear. Structural and biochemical studies have provided a clear explanation of the underlying mechanism for how the ExsE-ExsC and ExsD-ExsC interactions support signaling^14,15,38^. However, the mechanism whereby ExsD inhibits ExsA dimerization and DNA binding is still unknown. ExsD binds to the NTD of ExsA. Remarkably, this interaction does not involve either the canonical ligand binding site of AraC-type transcription factors or residues directly involved in ExsA dimerization^39,40^.

In the present study, we used structure-guided mutagenesis in conjunction with *in vitro* transcription measurements to map sections of the ExsD-ExsA interface. Our work not only pinpointed regions critical for ExsD-ExsA interaction, but also shed light onto the question of how ExsC liberates ExsA by sequestering ExsD.

## Results and Discussion

### Conserved surface exposed residues on ExsA are not crucial for ExsD binding

**Figure 1:**
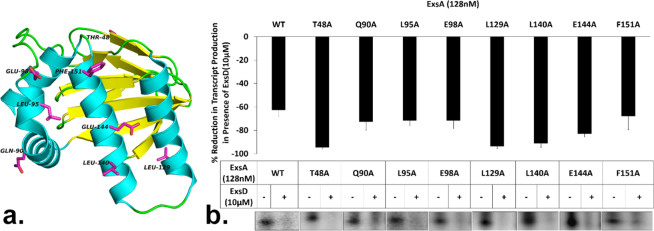
Conserved surface residues of ExsA are not critical for ExsD binding. (**a**) Structure of the ExsA-NTD wherein the targeted surface residues are highlighted. The figure was generated using Pymol (The PyMOL Molecular Graphics System, Version 2.0 Schrödinger, LLC). (**b**) Representative autoradiograms of three independent *in vitro* transcription experiments analyzing the susceptibility of ExsA variants containing substitutions in conserved surface residues to regulation by ExsD. All variants were sensitive to the presence of ExsD in the assay. Quantitative analysis of the autoradiograms was performed with ImageQTL. The bar diagrams show the averages obtained from three independent experiments with the error bars representing the standard deviations. . Gel image shown is representative of the results we obtained from three replicates.

We have previously shown that mutations in the putative ligand/ExsD binding pocket of ExsA, do not affect ExsD binding^39^ nor does ExsD appear to directly interact with residues that form the interface of the ExsA dimer^39^. Therefore, a broader approach was taken toward identifying residues of ExsA that are critical for ExsD binding. To limit the scope of the search, we initially targeted residues that are conserved among ExsA homologs. Due to the rarity of ExsD homologs and the plethora of AraC-like proteins, we first identified ExsD homologs and subsequently screened for the presence of an AraC-type protein in the same operon even though we were cognizant that *exsD* and *exsA* are not located in the same operon in *P*. *aeruginosa*. Seven different *exsD*-*exsA* pairs were identified (Fig. S1a,b). Initially, a set of eleven highly conserved ExsA residues with large solvent accessible surface areas were chosen for alanine substitutions: E37, T48, Q90, R91, L95, E98, R101, L129, L140, E144, and F151 **(**Fig. 1a). All variants expressed stably and could be readily purified **(**Fig. S2**)**. Eight of the ExsA variants, ExsAT48A, ExsAQ90A, ExsAL95A, ExsAE98A, ExsAL129A, ExsAL140A, ExsAE144A, and ExsAF151A, robustly activated *in vitro* transcription from an ExsA-dependent template. However, none of the eight studied mutations affected ExsD binding, demonstated by their susceptibility to inhibition by ExsD at levels comparable to those seen for wtExsA (Fig. 1b).

### Replacing amino terminal residues of ExsA with glycines affects ExsD binding but these residues are not required for the interaction

**Figure 2:**
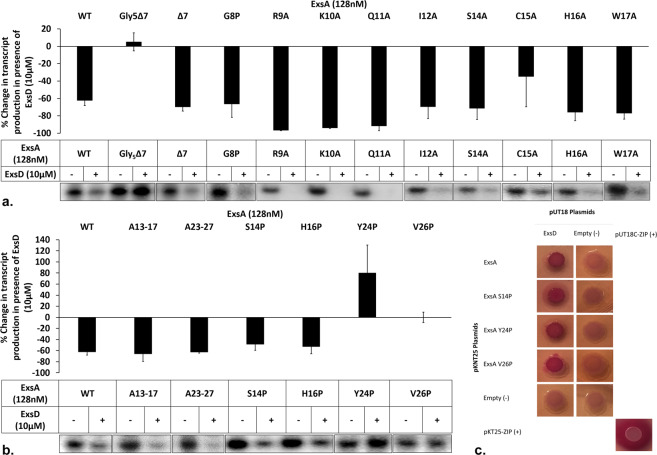
Backbone contacts between ExD and ExsA are critical for binding. (**a**) The Gly_5_∆7ExsA variant was insensitive to the presence of ExsD in the assay. However, ∆7ExsA protein behaved like wtExsA. Point substitutions in the periphery of the ExsA N-terminus showed no effect on ExsD regulation in our *in vitro* transcription assay. The bar diagrams show the averages obtained from three independent experiments with the error bars representing the standard deviations. The gel images shown are representative of the results we obtained from three replicates. (**b**) Residues in β-strands β-1 and β-2 of ExsA were replaced by stretches of alanines to determine if the side chains are important for ExsA binding. Both variants still respond to ExsD, demonstrating that the side chains of the substituted residues are not critical. However, proline substitutions of strand β-2 residues Y24 and the V26 impact ExsD dependent regulation. The bar diagrams show the averages obtained from three independent experiments with the error bars representing the standard deviations. The gel images shown are representative of the results we obtained from three replicates. (**c**) Results from a BACTH test demonstrate that, although ExsAY24P and ExsAV26P are insensitive to the regulation by ExsD, the two ExsA mutant still interact with ExsD, suggesting the presence of a larger ExsA-ExsD interface. Image shown is representative of the results we obtained from three biological replicates.

In the ExsA homolog AraC the amino terminus (N-terminus) interacts with the activator molecule arabinose. In this system arabinose binding induces a conformational change needed for binding of the AraC dimer to adjacent sites on the promoter^41^. We hypothesized that the amino-terminus of ExsA might also play a regulatory role. While the flexible N-terminal arm of AraC encompasses 26 residues, only the first 10 are unstructured in the crystal structure of the ExsA-NTD^39^. To test the hypothesis that this region might be involved in ExsD binding, we initially deleted the first seven codons of *exsA*. Because the ExsA variants were expressed as His_6_-MBP fusion proteins with a TEV protease cleavage site N-terminal to the native sequence, there was an initial concern that the cleavage site might not be accessible to the protease in the truncated protein. Therefore, we inserted five glycine residues C-terminal to the TEV protease cleavage site to create a His_6_-MBP-Gly_5_Δ7ExsA fusion protein. The purified Gly_5_Δ7ExsA protein promotes ExsA-dependent transcription at levels comparable to those observed for wtExsA. However, ExsD has no measurable effect on transcription activation by Gly_5_Δ7ExsA **(**Fig. 2a**)**.

To corroborate the direct role of the first seven amino acids of ExsA in the interaction with ExsD, we created a Δ7ExsA variant lacking the five glycine residues. The His_6_-MBP-Δ7ExsA fusion protein was readily processed by TEV protease and the purified Δ7ExsA protein successfully induced transcription from the ExsA-dependent promoter. However, contrary to the Gly_5_Δ7ExsA variant the function of Δ7ExsA *was* inhibited by ExsD, suggesting that the previously observed loss of inhibition in case of the Gly_5_Δ7ExsA variant was indirectly caused by the introduction of the five glycines (Fig. 2a). Because replacing the first seven ExsA residues with five glycines affected the interaction with ExsD, we reasoned that the actual interacting residues should be in spatial proximity of the ExsA N-terminus. We introduced nine single amino acid substitutions to create the G8P, R9A, K10A, Q11A, I12A, S14A, C15A, H16A, and W17A mutations in ExsA (Fig. 2a). The G8P variant was created to constrain the protein backbone and thus determine if the flexibility of the ExsA N-terminus is pivotal for ExsD binding. All nine ExsA variants induced transcript production from the ExsA-dependent promoter but none of the mutated residues proved critical for ExsA function because all variants also remained sensitive to ExsD **(**Fig. 2a**)**.

### The beta strand β2 of ExsA forms crucial backbone contacts with ExsD

In canonical AraC family enzymes the N-terminal regulatory domain forms a hollow beta-barrel structure containing the ligand binding pocket^34,42,43^. It was previously shown for ExsA that conserved pocket residues are not critical for ExsD binding^39^. Yet the crystal structure of ExsA-NTD indicates that the flexible N-terminus should be positioned near the beta barrel cavity. Because a modification at the N-terminus indirectly affects ExsD binding but no individual point mutations showed an effect, it was hypothesized that the beta barrel interacts with ExsD through the formation of an intermolecular beta-sheet. This hypothesis is particularly attractive because the competitive interactions of ExsD with the T3SS chaperone ExsC have previously been shown to involve intermolecular β-strand contacts^38^. Based on the ExsA-NTD crystal structure, strands β1 and β2 could bind ExsD with the amide groups of β1 residues Ser-14 and His-16 and β2 residues Tyr-24 and Val-26 oriented toward the outside. The four residues were individually replaced by prolines to block the amide groups in these positions and thus potentially interfere with ExsD binding. All four variants expressed stably and were tested in the *in vitro* transcription assay. While the β1 mutations had no impact, the ExsAY24P and ExsAV26P variants did not respond to ExsD in the assay **(**Fig. 2b**)**, suggesting that β2, but not β1, mediates contacts with ExsD. An attempt to create an ExsAY24P-V26P double mutant was unsuccessful because the protein was not stably expressed.

To determine if backbone contacts are the primary determinant for ExsD-ExsA binding, two additional ExsA variants were constructed. Here, β1 strand residues 13–17 and β2 residues 23–27 were respectively replaced with five alanine residues. In a prior study, stretches of alanine residues were shown to assume different secondary structures depending on the flanking residues and overall structural context^44^. Therefore, in the context of ExsA, the beta-barrel was expected to retain its structure despite the concurrent introduction of five amino acid substitutions each. Moreover, if only backbone contacts are pivotal for ExsD binding both variants should still be fully functional and remain sensitive to the presence of ExsD in the *in vitro* transcription experiment. Consistent with our model both proteins activated transcription from the ExsA-specific promoter, but transcription was inhibited by ExsD **(**Fig. 2b**)**, thus confirming that not the sequence *per se* but the secondary structure of ExsA β-2 is critical for effective ExsD binding.

In order to directly determine if the Y24P and V26P mutations fully disrupt ExsD-ExsA binding, we used a bacterial two-hybrid (BACTH) system. The selected BACTH system is based on the reconstitution of an adenylate cyclase from peptide fragments T18 and T25. These fragments, when brought in close proximity by the interacting proteins, catalyze cAMP synthesis. Because the N-termini of ExsD and ExsA are implicated in binding, the gene fragments of T18 and T25 were respectively fused to the 3′ ends of *exsD* and *exsA*. In the assay ExsA^Y24P^ and ExsA^V26P^ still interact with ExsD **(**Fig. 2c), suggesting that the strand-to-strand interactions between ExsD and ExsA are critical for proper positioning of ExsD on ExsA and do not encompass the entire interface.

### The N-terminal 20 residues of ExsD are important for inhibiting ExsA-dependent transcription

**Figure 3:**
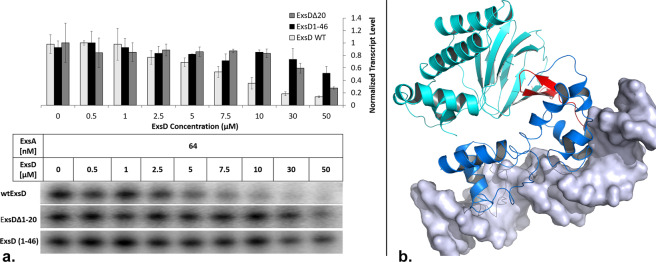
The Amino-terminus of ExsD is essential for the inhibition of ExsA-dependent transcription. (**a**) Results of an *in vitro* transcription assay comparing the concentration-dependent inhibition of *in vitro* transcription by three different ExsD constructs demonstrating that the ExsD N-terminus is essential but alone not sufficient for ExsD function. The bar diagrams show the averages obtained from three independent experiments with the error bars representing the standard deviations. Gel image shown is representative of the results we obtained from three replicates.. (**b**) Model of an ExsA-promoter (AraC-domain in marine, NTD in cyan, DNA shown as gray surface) complex with the ExsD N-terminal peptide (shown in red). The figure was generated using Pymol (The PyMOL Molecular Graphics System, Version 2.0 Schrödinger, LLC).

If strand-to-strand interactions form the foundation of effective ExsD-ExsA binding, this raises the question as to what region of ExsD is involved. In prior work on the ExsD-ExsC complex, the 46 N-terminal residues of ExsD were shown to be sufficient for ExsC binding. Sequence similarities between ExsD and ExsE suggest that, like ExsE, N-terminal residues of ExsD form a β-strand that interacts with the β-sheet of ExsC. Because binding of ExsD to either ExsC or ExsA is mutually exclusive we sought to determine if the N-terminus of ExsD is also involved in ExsA binding. Consistent with this model, an ExsDΔ20 variant was significantly attenuated in its ability to inhibit ExsA-dependent transcription^45^ (Fig. 3a). While the precise determination of an IC_50_ value was not possible due to the limits of ExsDΔ20 solubility a seven-fold higher ExsD concentration was needed compared to wild-type ExsD to reach 50% inhibition. In order to determine if the ExsD N-terminus is not only necessary but also sufficient for ExsA regulation, a synthetic polypeptide composed of the first 46 amino acids of the ExsD N-terminus (ExsD1–46) was purchased and examined in the *in vitro* transcription assay. ExsD1–46 did inhibit ExsA-dependent transcription, but only weakly **(**Fig. 3a**)**. This finding confirms our result from the BACTH assay, that the strand-to-strand interactions are pivotal but the entire interface involves additional regions on both proteins. In order to determine if the remainder of ExsD forms specific interactions with ExsA or merely provides sterically important bulk, we also created an ExsD_1-38_-MBP fusion protein and tested it in the *in vitro* transcription assay. This protein had no impact on ExsA-dependent transcription further confirming our prior finding that additional regions of ExsD are important for the specificity of ExsD-ExsA interactions (Fig. S3).

## Conclusion

ExsA, the central transcriptional activator for T3SS genes in *P*. *aeruginosa*, belongs to a subgroup of AraC-type transcription factors that are regulated by other proteins. Feedback loops, akin to the ExsACDE cascade that link activation of a central AraC-type transcription factor to host cell contact, have been identified in several other important pathogens, such as *Yersinia pestis*^46,47^, *Salmonella enterica*^48^, *Shigella flexineri*^49^, and *Vibrio parahaemolyticus*^50–53^. In fact, *V. parahaemolyticus* encodes homologs of all four proteins and they appear to function in the same manner as in *P*. *aeruginosa*^54^. The initial hypothesis that AraC proteins use the same site for interaction with their cognate ligands turned out to be incorrect^39^. In the same study, we also demonstrated that ExsD does not directly bind at the ExsA dimerization interface, which seemed a reasonable assumption as prior work had shown that ExsD prevents ExsA dimerization. The present work shows that the versatile N-terminus of ExsD not only mediates interactions with the T3SS chaperone ExsC but is also required for ExsA binding. Key for both interactions is the propensity of the ExsD N-terminus to form strand-to-strand interactions. What remains to be determined is how ExsD and ExsA create specificity for their interaction. Our data demonstrate that additional regions of ExsD are pivotal for ExsA binding, likely not only for enhancing binding affinity, but also to provide specificity. The specificity might be created by many low-affinity contacts, therefore a crystal structure of the ExsD-ExsA complex might be needed to elucidate the entire interface.

How ExsD mechanistically inhibits ExsA remains to be resolved. ExsD does not compete directly with ExsA dimerization. Instead ExsD may simply use its bulk to sterically interfere with homodimerization of ExsA. However, there is an alternative possibility. For the regulation of AraC and ToxT, interactions between their NTD and carboxy-terminal domain (CTD) are pivotal. In an AraC dimer the flexible N-terminus of one protomer interacts with the DNA-binding domain of the other AraC molecule to “lock” the AraC dimer in a conformation incapable of activating transcription. Binding of arabinose “unlocks” the dimer to induce transcription^41,55,56^. In ToxT, fatty acid binding to each molecule within the ToxT dimer actually locks the molecules into a rigid and inactive conformation. Here, not the N-terminus but the flexible inter-domain linker plays an important role in the regulatory process^33,34,56^. Unlike the case for AraC, the seven N-terminal residues of ExsA are dispensable. However, the ExsA dimer binds its promoters in a head-to-tail arrangement^57^, suggesting that the interdomain linker must be flexible to permit both the symmetric head-to-head dimerization of the NTDs and, at the same time, sequential head-to-tail binding of the CTDs to the promoter. We generated a model of an ExsA dimer using ToxT as the template. Interestingly, the N-terminus of ExsD would insert directly at the interface of the two ExsA domains (Fig. 3b**)**. Therefore, it is possible that, in line with what has been observed for ToxT and AraC, ExsD acts by targeting the contacts between the NTD and CTD of ExsA, thus forming a locked conformation of ExsA. More work will be needed to determine if there is a universal mechanism whereby AraC proteins are regulated.

## Methods

### Construction of wild-type ExsD and ExsA

Expression and purification of wild-type ExsD (wtExsD) and wild-type ExsA (wtExsA) followed previously published protocols^39,45^. Both proteins were respectively overexpressed as His_6_-MBP-ExsD and His_6_-MBP-ExsA fusions from the pFS-HMBPExsD and pFS-HMBPExsA vectors constructed by Gateway recombinational cloning (Invitrogen, Carlsbad, CA, USA)^58^.

### Construction of ExsD and ExsA variants

Construction of the ExsD^M59R^ variant has been described previously^45^. The 1–46 residue ExsD peptide (ExsD1-46) used in the experiments was purchased from New England Peptide, Inc. (Garner, MA, USA).

All ExsA variants were constructed with the QuikChange kit (Agilent Technologies, Santa Clara, CA, USA) using complementary primers with the appropriate base substitutions and pDONR201-wtExsA as template. The mutated genes were sequence-verified and recombined into the pDEST-His-MBP expression plasmid using Gateway recombinational cloning (Invitrogen). The list of primers used can be found in the Supplementary Table S1.

### Purification of ExsD and ExsA proteins

Expression and purification of all ExsD and ExsA variants followed previously described protocols^39,45^ with the exception that ExsA variant expression was induced at 14 °C instead of 18 °C. ExsDΔ1-20 was obtained by performing limited proteolysis of wtExsD using thermolysin, as previously described^59^.

### ExsA-dependent *in vitro* transcription assay

This details of the assay have been published previously^39,45^. Briefly, the P_*exsD*_ promoter template encompasses positions −207 to 94 of the promoter from which RNA polymerase produces an 82 base mRNA transcript in presence of wtExsA. The P_*exsD*_ template was produced by PCR using the following primers: 5′-CATCAGTTGCTGCTCAACAGCG-3′ and 5′-CACCGCTTCTCGGGAGTACTGC-3′. PCR products were run on a 2% agarose gel and purified using the Wizard SV Gel and PCR Clean-up System (Promega, Madison, WI, USA). Each 30 μL transcription assay reaction contained 4.4 fM of promoter template, 2 U *E*. *coli* RNA polymerase holoenzyme (New England Biolabs Inc., Ipswich, MA, USA), 1 U RiboGuard RNase Inhibitor (Epicentre Biotechnologies, Madison, Wisconsin, USA), 15 ng/μL poly(deoxyinosinic-deoxycytidylic) acid (to prevent non-specific transcription initiation), 133 mM NaCl, 32 mM Tris-HCl (pH 7.4), 10 mM MgCl_2_, 25 μM EDTA, 0.9 mM TCEP, 0.2 mM DTT, 50 μM BSA, and 15.5% glycerol. Each experiment contained 128 nM wtExsA. Samples were mixed and allowed to equilibrate at room temperature for five minutes. To start the reaction, 3 μL NTPs (stock concentrations of 200 μM ATP, CTP, GTP, and 40 μM UTP) mixed with 0.2 μL (0.2 μCi) of 3.3 mM α-32P UTP was added to each sample. Reactions were incubated at 37 °C for 10 minutes. The reaction was terminated through the addition of 12 μL stop solution containing 3 M ammonium acetate, 50 mM EDTA, and 0.11 mg/mL glycogen. RNA was precipitated by adding 160 μL of ice cold ethanol and incubating the samples at −20 °C for one hour. Samples were then centrifuged for 15 minutes at 12,000 x g and the supernatants discarded. Pellets were resuspended in 12 μL of 1X TBE-urea sample buffer (Bio-Rad Laboratories, Inc., Hercules, California, USA) and incubated at 70 °C for five minutes. After a two-minute centrifugation, the samples were loaded onto a 10% TBE-urea gel and run at 200 mV for 60 min. Gels were exposed to a storage phosphor screen (GE Healthcare, Chicago, IL, USA) for 16 hr. The phosphor screen was scanned using a Typhoon Trio Variable Mode Imager (GE Healthcare), and gel bands were quantified using the Image Quant TL v2005 software package (GE Healthcare). Each experiment was performed in triplicate. The images in the manuscript constitute composites made from the original gel images that are shown in Figs. S3 to S13.

### Bacterial adenylate cyclase two-hybrid (BACTH) assay

The BACTH assay measures the ability of adenylate cyclase to synthesize cAMP, which provides an output to assess binding between target proteins. Adenylate cyclase has been split into two fragment peptides T18 and T25, which must be in direct contact for cAMP synthesis to occur^60,61^. The T18 and T25 DNA sequences are fused to the genes encoding target proteins in separate plasmids and co-expressed in *cya E*. *coli* strain BTH101. Activation of cAMP synthesis, indicating physical interaction between the target proteins, was visualized on MacConkey agar via the change in colony color from clear to bright pink when the proteins of interest interact. The interaction of wtExsA and wtExsD served as a positive control. The T18 fragment is expressed from high copy number pUT18 vector, and the T25 fragment is expressed from the low copy number pKNT25 vector. Both *exsA* and *exsD* were fused to the 5′-ends of the fragment genes in the pKNT25 and the pUT18 vectors, respectively and co-transformed into chemically competent BTH101. BTH101 was also co-transformed with vectors (pUT18C-zip and pKT25-zip) to serve as an additional positive control for the assay. Co-transformation of the empty pKNT25 and pUT18 vectors into BTH101 served as a negative control. Further negative controls involved co-transformation of the empty pKNT25 vector with the pUT18-wt*exsD* plasmid, as well as co-transformation of all *exsA* expressing constructs with the empty pUT18 vector. After growing the cells overnight in LB under ampicillin (100 μg/ml) and kanamycin (50 μg/ml) selection at 37 °C, the bacteria were plated on MacConkey agar plates and incubated at 30 °C for 24 hours. Transformants expressing interacting proteins cause increased production of β-galactosidase, which in turn leads to the development of a bright pink color. The lack of an interaction, on the other hand, results in colorless colonies on the indicator plates.

## Molecular Modeling

The model of an ExsA-promoter complex was created by superposing the structure of ExsA-NTD (PDB access code: 4ZUA) onto the NTD of the ToxT structure (PDB access code: 3GBG) (RMSD = 3.1 Å) and by superposing the MarA-DNA complex structure (PDB access code: 1BLO) onto the AraC-domain of ToxT (RMSD = 1.9 Å). The sequence of the N-terminus residues of ExsD was threaded onto the homologous region of ExsE and then manually placed by the β-2 strand of ExsA-NTD.

## Supplementary information

Supplemental information.
