# Supplementary material for: Backbone Interactions Between Transcriptional Activator ExsA and Anti-Activator ExsD Facilitate Regulation of the Type III Secretion System in Pseudomonas aeruginosa
Source: Sci Rep. 2020 Jun 18;10:9881. doi: 10.1038/s41598-020-66555-z (PMC7303211; doi:10.1038/s41598-020-66555-z)
Supplement: Supplementary file 1 — Supplemental information. [file 41598_2020_66555_MOESM1_ESM.pdf]

Backbone Interactions Between Transcriptional Activator ExsA and Anti-Activator ExsD Facilitate Regulation of the Type III Secretion System in *Pseudomonas aeruginosa*

Manisha Shrestha[1,2], Robert C. Bernhards[1,3,4], Yichen Fu[1,5], Kylie Ryan[1], and Florian D. Schubot[1*]

Author Affiliations
1 Department of Biological Sciences, Virginia Polytechnic Institute & State University, Washington Street, Blacksburg, VA 24060, USA
2. Department of Radiation Oncology, UT Southwestern Medical Center, Dallas, TX 75390, USA
3 Defense Threat Reduction Agency, Fort Belvoir, VA 22060, USA
4 U.S. Army Combat Capabilities Development Command (CCDC) Chemical Biological Center, Aberdeen Proving Ground, MD 21010, USA
5. College of International and Extended Studies, San Jose State University, San Jose, CA 95310, USA

***Corresponding author: E-mail: fschubot@vt.edu**

Supplementary Figures and Tables

Table S1. List of primers used to construct $ExsD_{1-38}$-MBP variant

6HisTEVExs D1-18r	CCTGCCAGCGAACACCGCTTCTCGGGAGTACTGCTTATCGTCTTCCTGCTCCA TTCCCCCCTGGAAGTACAGGTTCTCGTGATGGTGATGGTGATG
ExsD1-38MBP1-8f	GCG GTG TTC GCT GGC AGG CGG GTA TCC GTG GTG GGC TCG GAC GCC CGC TCG CGG GGT CGG GTG CCG GGT TAC GCA TCG **GCA** GAA GAA GGT AAA CTG GTA
5’ extension	CATATGGGCAGCCATCACCATCACCATCAC
3’ extension	ATCGCCGTTAATCCAGATTACCAGTTTACC

Table S2. List of primers used to construct variants of ExsA

E37A-fwd	GTATATGTTCTGCTCGCGGGCGAACTGACCGTC
E37A-rev	GACGGTCAGTTCGCCCGCGAGCAGAACATATAC
T48A-fwd	GTCCAGGACATCGATTCCGCTTTTTGCCTGGCGCCTG
T48A-rev	CAGGCGCCAGGCAAAAAGCGGAATCGATGTCCTGGAC
Q90A-fwd	GTTTCTACAAGGCTTCGTCGCACGCTTCGGCGCGCTGTTG
Q90A-rev	CAACAGCGCGCCGAAGCGTGCGACGAAGCCTTGTAGAAAC
R91A-fwd	CTACAAGGCTTCGTCCAGGCATTCGGCGCGCTGTTGAGTG
R91A-rev	CACTCAACAGCGCGCCGAATGCCTGGACGAAGCCTTGTAG
L95A-fwd	CCAGCGCTTCGGCGCGGCGTTGAGTGAAGTCGAG
L95A-rev	CTCGACTTCACTCAACGCCGCGCCGAAGCGCTGG
E98A-fwd	CGCTGTTGAGTGCAGTCGAGCGTTG
E98A-rev	CAACGCTCGACTGCACTCAACAGCG
R101A-fwd	GTTGAGTGAAGTCGAGGCATGCGACGAGCCCGTGC
R101A-rev	GCACGGGCTCGTCGCATGCCTCGACTTCACTCAAC
L129A-fwd	GGGGTTGAAGGAATTGGCTGTGCATGAGCATCCG
L129A-rev	CGGATGCTCATGCACAGCCAATTCCTTCAACCCC
E144A-fwd	CTGCCTGAAGATCGAGGCGTTGCTGATGCTCTTC
E144A-rev	GAAGAGCATCAGCAACGCCTCGATCTTCAGGCAG
L140A-fwd	CATCCGCCGATGCTCGCCTGCGCAAAGATCGAGGAGTTGCTGATG
L140A-rev	CATCAGCAACTCCTCGATCTTTGCGCAGGCGAGCATCGGCGGATG
F151A-fwd	GTTGCTGATGCTCTTCGCGGCAAGTCCGCAGGGGCCGCTG
F151A-rev	CAGCGGCCCCTGCGGACTTGCCGCGAAGAGCATCAGCAAC
TevExsA2-7GlyFwd	GAGAACCTGTACTTCCAGGGTGGTGGTGGAGGAGGCCGAAAGCAGATAACGTCT
TevExsA2-7GlyRev	AGACGTTATCTGCTTTCGGCCTCCTCCACCACCACCCTGGAAGTACAGGTTCTC
ExsA N27F-rev	CATATACGCCCTCTTCCTTGAATACCCTGTATTCGAAAGTTG
ExsAT48R-fwd	CAGGACATCGATTCCAGGTTTTGCCTGGCGCCTG

ExsA T48R-rev	CAGGCGCCAGGCAAAACCTGGAATCGATGTCCTG
ExsA L95R-fwd	CGCTTCGGCGCGCGGTTGAGTGAAGTC
ExsA L95R-rev	GACTTCACTCAACCGCGCGCCGAAGCG
ExsA E98F-fwd	CGGCGCGCTGTTGAGTTTCGTCGAGCGTTGCGACG
ExsA E98F-rev	CGTCGCAACGCTCGACGAAACTCAACAGCGCGCCG
ExsA L129R-fwd	GGGTTGAAGGAATTGCGTGTGCATGAGCATCCG
ExsA L129R-rev	CGGATGCTCATGCACACGCAATTCCTTCAACCC
ExsA L140R-fwd	GATGCTCGCCTGCCGGAAGATCGAGGAGT
ExsA L140R-rev	ACTCCTCGATCTTCCGGCAGGCGAGCATC
ExsA E144F-fwd	GCCTGCCTGAAGATCGAGTTCTTGCTGATGCTCTTCGCG
ExsA E144F-rev	CGCGAAGAGCATCAGCAAGAACTCGATCTTCAGGCAGGC
ExsA F151R-fwd	TGATGCTCTTCGCGCGCAGTCCGCAGGGGC
ExsA F151R-rev	GCCCCTGCGGACTGCGCGCGAAGAGCATCA
Δ7ExsA-fwd	GAGAACCTGTACTTCCAGGGCCGAAAGCAGATAACGTCT
Δ7ExsA-rev	AGACGTTATCTGCTTTCGGCCCTGGAAGTACAGGTTCTC
Δ5ExsA-fwd	AACCTGTACTTCCAGTCTCTTGGC
Δ5ExsA-rev	GCCAAGAGACTGGAAGTACAGGTT
Δ6-10ExsA-fwd	CAAGGAGCCAAAATAACGTCT
Δ6-10ExsA-rev	AGACGTTATTTTGGCTCCTTG
Q2A ExsA-fwd	GGAGAACCTGTACTTCCAGGGTGCAGGAGCCAAATC
Q2A ExsA-rev	GATTTGGCTCCTGCACCCTGGAAGTACAGGTTCTCC
K5AExsA-fwd	CTTTCGGCCAAGAGATGCGGCTCCTTGACCCTGG
K5AExsA-rev	CCAGGGTCAAGGAGCCGCATCTCTTGGCCGAAAG
R9AExsA-fwd	CAAGACGTTATCTGCTTTGCGCCAAGAGATTTGGCTCC
R9AExsA-rev	GGAGCCAAATCTCTTGGCGCAAAGCAGATAACGTCTTG
K10AExsA-fwd	TGACAAGACGTTATCTGCGCTCGGCCAAGAGATTTGGCTC
K10AExsA-rev	GAGCCAAATCTCTTGGCCGAGCGCAGATAACGTCTTGTCA
Q11AExsA-fwd	CCAATGACAAGACGTTATCGCCTTTCGGCCAAGAGATTTG
Q11AExsA-rev	CAAATCTCTTGGCCGAAAGGCGATAACGTCTTGTCATTGG
ExsAd11F	GAGAACCTGTACTTCCAGGGCATAACGTCT
ExsAd11R	AGACGTTATGCCCTGGAAGTACAGGTTCTC
ExsAd20F	GAGAACCTGTACTTCCAGGGCACTTTCGAA
ExsAd20R	TTCGAAAGTGCCCTGGAAGTACAGGTTCTC
ExsAG8Pf	CGTTATCTGCTTTCGGGGAAGAGATTTGGCTCCTTGGCC
ExsAG8Pr	GGCCAAGGAGCCAAATCTCTTCCCCGAAAGCAGATAACG
ExsAI12Af	ATCTCTTGGCCGAAAGCAGGCAACGTCTTGTCATTGGAACA
ExsAI12Ar	TGTTCCAATGACAAGACGTTGCCTGCTTTCGGCCAAGAGAT
ExsAS13Af	GGCCGAAAGCAGATAACGGCTTGTCATTGGAACATTC
ExsAS13Ar	GAATGTTCCAATGACAAGCCGTTATCTGCTTTCGGCC
ExsAC15Af	TGGCCGAAAGCAGATAACGTCTGCTCATTGGAACATTCCAA
ExsAC15Ar	TTGGAATGTTCCAATGAGCAGACGTTATCTGCTTTCGGCCA

ExsAH16Af	CGAAAGCAGATAACGTCTTGTGCTTGGAACATTCCAACTTTCGA
ExsAH16Ar	TCGAAAGTTGGAATGTTCCAAGCACAAGACGTTATCTGCTTTCG
ExsAW17Af	GAAAGCAGATAACGTCTTGTCATGCGAACATTCCAACTTTCGAATACA
ExsAW17Ar	TGTATTCGAAAGTTGGAATGTTCGCATGACAAGACGTTATCTGCTTTC
ExsA2_7alaf	GAGAACCTGTACTTCCAGGGTGCAGCAGCCGCAGCTGCTGGCCGAAAGCAGATAACGTCT
ExsA2_7alar	AGACGTTATCTGCTTTCGGCCAGCAGCTGCGGCTGCTGCACCCTGGAAGTACAGGTTCTC
ExsA13_17alaf	TCTCTTGGCCGAAAGCAGATAGCGGCTGCTGCTGCGAACATTCCAACTTTCGAATAC
ExsA13_17alar	GTATTCGAAAGTTGGAATGTTCGCAGCAGCAGCCGCTATCTGCTTTCGGCCAAGAGA
ExsA23_27alaf	CATTGGAACATTCCAACTTTCGCAGCCGCGGCAGCCAAGGAAGAGGGCGTATATGTT
ExsA23_27alar	AACATATACGCCCTCTTCCTTGGCTGCCGCGGCTGCGAAAGTTGGAATGTTCCAATG
ExsAT13P_f	CTTGGCCGAAAGCAGATACCGTCTTGTCATTGG
ExsAT13P_r	CCAATGACAAGACGGTATCTGCTTTCGGCCAAG
ExsAR25P_f	CCAACTTTCGAATACCCGGTAAACAAGGAA
ExsAR25P_r	TTCCTTGTTTACCGGGTATTCGAAAGTTGG
S14Pfwd	GGCCGAAAGCAGATAACGCCTTGTCATTGGAACATTC
S14Prev	GAATGTTCCAATGACAAGGCGTTATCTGCTTTCGGCC
H16Pfwd	AAAGCAGATAACGTCTTGTCCTTGGAACATTCCAACTTTCG
H16Prev	CGAAAGTTGGAATGTTCCAAGGACAAGACGTTATCTGCTTT
Y24Pfwd	GTCATTGGAACATTCCAACTTTCGAACCCAGGGTAAACAAGGAAGAG
Y24Prev	CTCTTCCTTGTTTACCCTGGGTTCGAAAGTTGGAATGTTCCAATGAC
V26Pfwd	TCCAACTTTCGAATACAGGCCAAACAAGGAAGAGGGCGTA
V26Prev	TACGCCCTCTTCCTTGTTTGGCCTGTATTCGAAAGTTGGA
Y24Afwd	GTCATTGGAACATTCCAACTTTCGAAGCCAGGGTAAACAAGGAAGAG
Y24Arev	CTCTTCCTTGTTTACCCTGGCTTCGAAAGTTGGAATGTTCCAATGAC
V26Afwd	AACTTTCGAATACAGGGCAAACAAGGAAGAGGGCG
V26Arev	CGCCCTCTTCCTTGTTTGCCCTGTATTCGAAAGTT

Fig. S1. a. Sequence identifier and alignment of ExsD from different bacteria

Q9I321_PSEAE ExsD	*Pseudomonas aeruginosa*
Q699Q3_AERHY ExsD	*Aeromonas hydrophila*
A5Y876_9GAMM ExsD	*Aeromonas veronii*
B6VK47_ PHOAA ExsD	*Photorhabdus asymbiotica subsp. Asymbiotica*
A4SUG1_AERS4 ExsD	*Aeromonas salmonicida*
Q7N0V3_PHOLL Uncharacterized protein	*Photorhabdus luminescens subsp. laumondii*
Q87P20_VIBPA Uncharacterized protein	*Vibrio parahaemolyticus serotype O3:K6*

```
Q9I321 ---MEQEDDKQYSREAVF-----AGRRVSVVGSDARSRG-------RVPGYASSSLYRES 45
Q699Q3 ---MSQ-QDHNSSNQGLF-----AGRRVTVVQPDTLSRD-------RLVGQLSVLRYQDA 44
A5Y876 ---MSQ-QDHHSPNQGLF-----AGRRVTVVQPDTLSRD-------RLVGQLSVLRYQDA 44
B6VK47 ---MSQ-QDHNQTRTSMF-----LGRKISVMQSGIPRRD-------QLLGKSQPAQSQNT 44
A4SUG1 ---MSQ-QDHHSPNQGLF-----AGRRVTVVQPDTLSRD-------RLVGQLSVLRYQDA 44
Q7N0V3 ---MSQ-QDHNQTRTSMF-----LGRKISVTQSGIPRRD-------QLLGKSSAIQYQNR 44
Q87P20 MRRRTQMKKQHWRRRSLFPDSIVTQRKVTVLQRGARYESASQPLQDLNVVHVNHRQLLSE 60
            * ..::  . .:*       *:::*   .   ..             .     .

Q9I321 GIISARQLALLQRMLPRLRLEQLFRCEWLQQRLARGLALGREEVRQILLCAAQDD----- 100
Q699Q3 GVITSQQMDLLQRLLPRTRLESLLESLWFQRRLDAALSVSREELQQILRLAGSER----- 99
A5Y876 GVITSQQMALLQRLLPRTRLESLLGSIWFQRRLDAALAVSREELQQILRLAGSER----- 99
B6VK47 GIINSQQSVLLQRLLPRRRLESLTKSVWFHRRLNCGQTLRRDELQQLIRTAAKPE----- 99
A4SUG1 GVITSQQMALLQRLLPRTRRESLLGSIWFQRRLDAALAVSREELQQILRLAGSEH----- 99
Q7N0V3 DIINSQQFILLQRLLPRHLLESLMKSVWFRRRLNCGQALRRDDLQQLIRAAAELE----- 99
Q87P20 GVLNDDQLSLLQRLLDRSVVDSLCASQLVKTYLRLGTSIDRFAMRLFLEIGAQLSDSQRV 120
       .::.  *  ****:* *   :.*  .  .:  *  . :: *  :: ::  ...

Q9I321 -------DGWCSELGDRVNLAVPQSMIDWVLLPVYGWWESLLDQAIPGWRLSLVELETQS 153
Q699Q3 -------YDWLQQLGDRINLADRALLWHWVLHPLHRWWVQRLEPLYGAWRNELVQLQVMR 152
A5Y876 -------CDWMQQLGDRINLADRPLLWHWVLYPLHRWWVQRLEPLYGAWLNELEQLQVMR 152
B6VK47 -------CDWNQILGDHINLADKCLLQHWVLQPLFNWWLRLLEPEIELWFTELEQLQIQE 152
A4SUG1 -------CDWVQQLGDRINLADRPLLWHWVLYPLHRWWVQRLEPLYGAWLNELEQLQVMH 152
Q7N0V3 -------CDWNKVLGDRINLADRHLLQHWVLQPLFDWWLRLLEPEIDFWFTEFDQLQIQE 152
Q87P20 ATFEQRLEYINSRLGFRFNLATPKTLILCCYLALTEWIHRQTDQSALH---ASVKVEQLM 177
                  . ** :.***    :       :  *     :           :::

Q9I321 RQLRVKSEFWSRVAELEP--EQAREELARVAKCQARTQEQVAELAGKLETA--------- 202
Q699Q3 RQLNAQAVFWQTVVDVPA--DLESRIT----DQLAQLSQREQELTQLHSDC--------- 197
A5Y876 RQLNAQAVFWQTVVDVPA--DLESRIA----DQLEQLNQREQELTRLQSDC--------- 197
B6VK47 RQLHAKAHFWQQAENVPP--QCREQHQQEVISLQATLNQRKHQLEKQLVTT--------- 201
A4SUG1 RQLNARAVFWQTVVDVPA--DLESRIA----DQLEQLNQREQELTRLQSDC--------- 197
Q7N0V3 RQLHAKAHFWQQAESVSS--QCREQQHKEVTSLQTALNQRKQLLEKRLLTT--------- 201
Q87P20 NQLDIQKEYWSKLSGEDTSAIFVEQQLALIESQQTQLKAQLNTLNEQQSQVIESHKALVD 237
       .**  :  :*.             .      .     . :   *

Q9I321 ------------------SALAKSAWPNWQRGMATLLASGGLAGFEPIPEVLECLWQPLC 244
Q699Q3 ------------------EARLQLAWPAWYGQT---SQEGDPALLMPVPLELGVFWHALL 236
A5Y876 ------------------EARLQLAWPAWYAQT---SKEGDPVHLMPVPLELGTFWHALQ 236
B6VK47 ------------------ETHAREAWPNWFIGLDALQSDGNLMAFMPVPEALSSCWAWLT 243
A4SUG1 ------------------ETRLQLAWPAWYAQT---SKEGDLAHLMPVPLELGTFWHALQ 236
Q7N0V3 ------------------ETQAREAWPNWFTGLDALQTGGDLTAFMPVPEALSSCWAWLT 243
Q87P20 KWQPSLSNLKELADYTSTTDMFISDWKTWCSEAR------------LQAPDLNEVWDACD 285
                                *  *                      *   *

Q9I321 RLDDDVGAADAVQAWLHERNLCQAQDHFYWQS-------------- 276
Q699Q3 ALPHQDDVALTLHEWLVGRGIALGQDHFYWQPAEP----------- 271
A5Y876 ALPHQDEAALTLHEWLAGRGIALGQDHFYWQPPAP----------- 271
B6VK47 AIEYDSKAANHLQQWLCARALCLPQDSFHWQSTMA----------- 278
A4SUG1 ALPHQDEAALTLHEWLAGRGIALGQDHFYWQPPAP----------- 271
Q7N0V3 TLEHDTEAATHLQKWLCARFLCLPQDNFHWQSTT------------ 277
Q87P20 VVYNDLNAVAKVWQWFKDMQIVGDVDHYYFDIQSGQCGQACNHLSQI 332
        :  :  ..  :  *:    :    * ::::
```

b. Sequence identifier and alignment of ExsA from different bacteria

ExsA Sequence Identifier	Microorganism
EXSA_PSEAE	*Pseudomonas aeruginosa*
A4SUG2_AERS4	*Aeromonas salmonicida*
Q699Q4_AERHY	*Aeromonas hydrophila*
A5Y875_9GAMM	*Aeromonas veronii*
Q7N0V4_PHOLL	*Photorhabdus luminescens subsp. laumondii*
Q87P19_VIBPA	*Vibrio parahaemolyticus serotype O3:K6*
B6VK46_PHOAA	*Photorhabdus asymbiotica subsp. Asymbiotica*

```
EXSA_PSEAE               1   --MQGA---KSLGRK--QITSCHWNIPTFEYRVNKEEGVYVLLEGELTVQDIDSTFCLAP   53
A4SUG2_AERS4             1   --MKGI---TTTEKG--DMALLQWRMSAFNVIEHPQKGIYILLEGSMKWQDCTDTYELSP   53
Q699Q4_AERHY             1   --MNGI---TTAEKG--DMALLQWCMSAFNVIEHPQEGIYILLEGSITWQDCTDTYELTP   53
A5Y875_9GAMM             1   --MKGI---TTTEKG--DMALLQWCMSAFNVIEHPQEGIYILLEGSMKWQDCTDTYELSP   53
Q7N0V4_PHOLL             1   --MNGI---TTSGKGM-DISRLQRSMPAFNVIEHPQEGIYILLEGEMTWQDSTNTYDISH   54
Q87P19_VIBPA             1   MDVSGQLNTETVGSSLRKIRSFSHYEKHDEVFHSDQSHIVVVHNGQLRVQTGDCTIDVVA   60
B6VK46_PHOAA             1   --MNGI---TTSGKGM-DITLFQSSMPAFNVIEHHQEGIYILLEGEMTWQDSTNSYDISH   54
                               :.*     :      .:          :     :. : :: :*.:  *    :  :

EXSA_PSEAE              54   GELLFVRRGSYVVST--KGKDSRILWIPLSAQFLQGFVQRFGALLSEVERCDEPVPGIIA   111
A4SUG2_AERS4            54   NELLFVRPGNYAVCT--GDGPCRLLWLPLPNSFLQGFLQRFGSLLSEVPRLEGMAPTLLP   111
Q699Q4_AERHY            54   NQLLFVRRGNYAVCT--AGSPCRLLWLPLSNSFLQGFLQRFGSLLSEVARLEGMAPTLLP   111
A5Y875_9GAMM            54   NELLFVRRGNYAVCT--GDGPCCLLWLPLSNSFLQGFLQRFGSLLSEVPRLEGMAPTLLP   111
Q7N0V4_PHOLL            55   NELLFVRRGSYAAKT--RSDTCKLLWLPLSTTFLHGFLQRFGTLLSEVERHNMSTPELIA   112
Q87P19_VIBPA            61   GSGVFLSQGDYLLEYSPQGGNYHSSIIEFDNELVSQLLQKHSDLLMTLPKVDKLNSGLFS   120
B6VK46_PHOAA            55   NELLFVRRGSYVAKT--RSDTCKLLWLPLSTPFLHGFLQRFGTLLSEVERHNVPAPELIA   112
                             .. :*:  *.*       .       : :   ::  ::*:.. **  : : :     ::

EXSA_PSEAE             112   FAATPLLAGCVKGLKELLVHEHPPMLACLKIEELLMLFAFSPQGPLLMSVLRQLSNRHVE   171
A4SUG2_AERS4           112   FHSSPLLTQCIQGLLGLTEYEHPPALAQLRTEELLFLLAFGEQGPQLMSILRQLSNRQVE   171
Q699Q4_AERHY           112   FHSSPLLTQCIQGLYGLIDHEHPPALAQLRTEELLFLLAFGEQGPQLMSILRQLSNRQVE   171
A5Y875_9GAMM           112   FHSSPLLTQCIQGLRGLTEHEHPPALAQLRTEELLFLLAFGEQGPQLMSILRQLSNRQVE   171
Q7N0V4_PHOLL           113   FTSSPLLSQSINGLVDLLAHDYPTALGQLRTEELLLLLAFGEQGTLLMSVLRQLSNRQVE   172
Q87P19_VIBPA           121   FGLNILIEQVLSGMKTLEEQSYPDAIMRLKYEEMLILLLHSQGGEVLYALLSQQTNRTSD   180
B6VK46_PHOAA           113   FTSSPLLSQSINGLVDLLAHDYPTALGQLRTEELVLLMAFGEQGALLMSVLRQLSNRQVE   172
                             *  . *:   :.*:  *   .:*  :  *: **:::*: ..  *  * ::* * :**  :

EXSA_PSEAE             172   RLQLFMEKHYLNEWKLSDFSREFGMGLTTFKELFGSVYGVSPRAWISERRILYAHQLLLN   231
A4SUG2_AERS4           172   RLQQFMEKHYLMEWKLSEFAKEFGMGLTTFKELFGSIYGVSPRAWISERRILFAHQLLLN   231
Q699Q4_AERHY           172   RLQQFMEKHYLMEWKLSEFSKEFGMGLTTFKELFGSIYGVSPRAWISERRILFAHQLLLN   231
A5Y875_9GAMM           172   RLQQFMEKHYLMEWKLSEFAKEFGMGLTTFKELFGSIYGVSPRAWISERRILFAHQLLLN   231
Q7N0V4_PHOLL           173   RLQSFMENHYLKEWKLSEFAKEFGMGLTTFKELFRSVYGISPRAWISERRILYAHQLLLN   232
Q87P19_VIBPA           181   RLRRFMEQHYLKEWKLTDYAQEFGASLTTFKELFNEHYGISPRAWISERRLLHAHKLLLT   240
B6VK46_PHOAA           173   RLQIFMENHYLKEWKLSEFAKEFGMGLTTFKELFRSVYGISPRAWISERRILYAHQLLLN   232
                             **: ***:*** ****:::::*** .******** . **:**********:*.**:***.

EXSA_PSEAE             232   SDMSIVDIAMEAGFSSQSYFTQSYRRRFGCTPSRSRQGKDECRAKNN   278
A4SUG2_AERS4           232   SEMSIVDIAMEAGFSSQSYFTQSYRRRFGCTPSRARHGTD-------   271
Q699Q4_AERHY           232   SPSSIVDIAMEAGFSSQSYFTQSYRRRFGCTPSRARHGTD-------   271
A5Y875_9GAMM           232   SEMSIVDIAMEAGFSSQSYFTQSYRRRFGCTPSRARHGTD-------   271
Q7N0V4_PHOLL           233   SEMSIVDISMEAGFSSQSYFTQSYRRRFNCTPSRARYGKE-------   272
Q87P19_VIBPA           241   SKMSIVDVAMEAGFSSQSYFTQSYRRRFGTTPSKVRSGDEQVAIAN-   286
B6VK46_PHOAA           233   SEMSIVDISMEAGFSSQSYFTQSYRRRFDCTPSRARYGKE-------   272
                             *  ****::*******************. ***: * * :
```

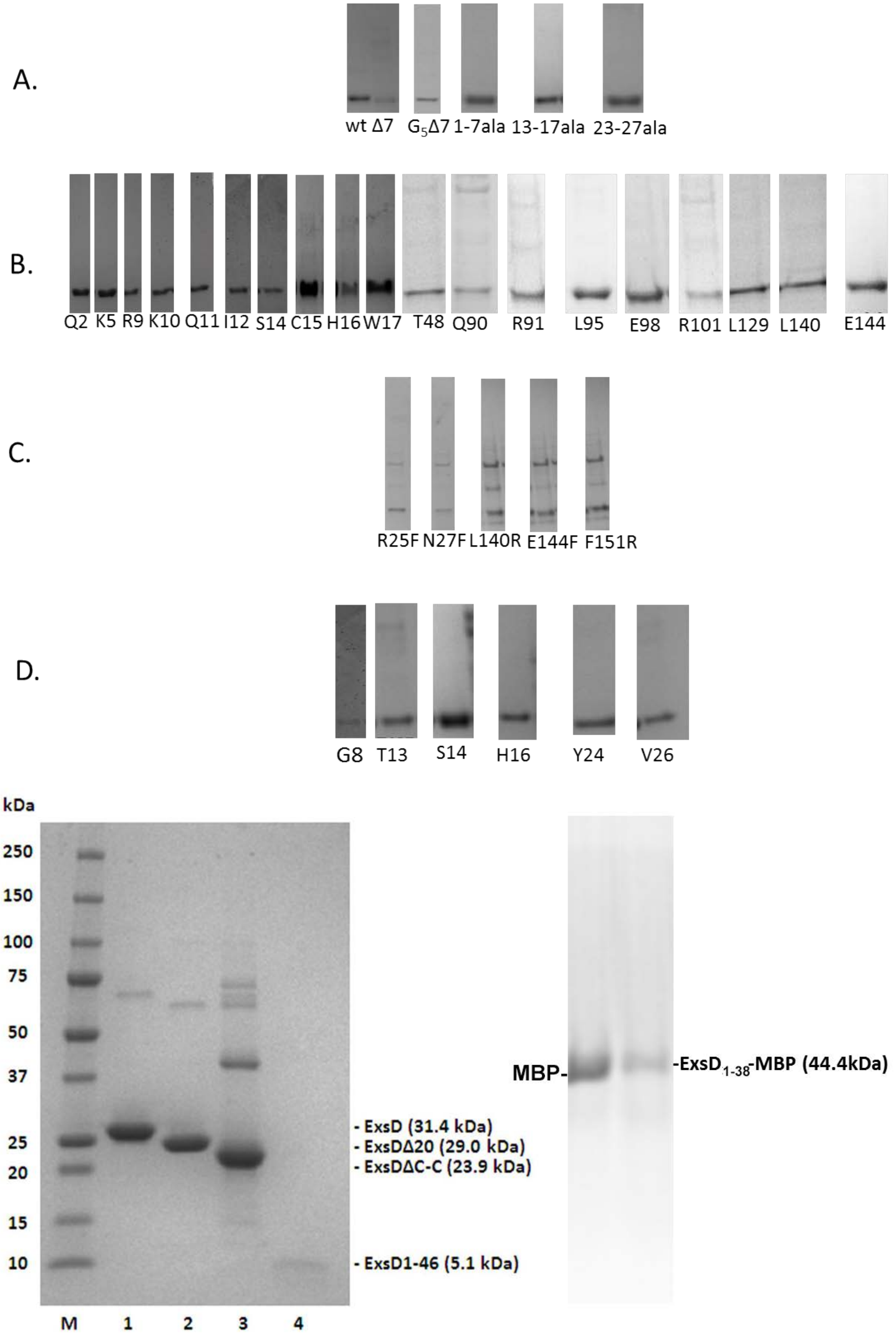

S2. Coomassie stained gels of variants of ExsA and ExsD. a. Amino-terminal regions modified ExsA. Panel **B**. Alanine substituted variants of ExsA. **C.** Disruptive substitution variants of ExsA. **D.** Proline substituted variants of ExsA. E. Different variants of ExsD.

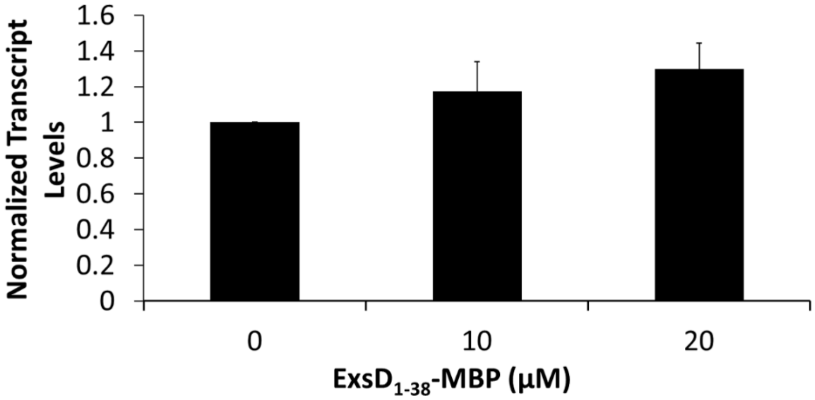

ExsA (nM)	-	128		
ExsD$_{1-38}$-MBP (μM)	-	0	10	20

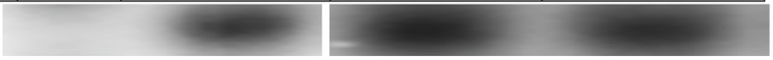

S3. Results of *in vitro* transcription experiments testing if an ExsD$_{1\text{-}38}$-MBP fusion protein can inhibit ExsA-dependent transcription. No significant inhibition was observed. Each experiment was performed in triplicate and the error bars represent the standard error of the independent reactions. The uncropped image is given in S14.

Original Gel Images: The gel images generated from the *in vitro* transcription experiments reported in the three figures of the manuscript have been cropped and assembled into composite gel images. The following are the original gel images. The ExsA-dependent templates are framed and the lanes use in the final manuscrpt are labeled.

Images associated with Figures 1 and 2 in the Manuscript

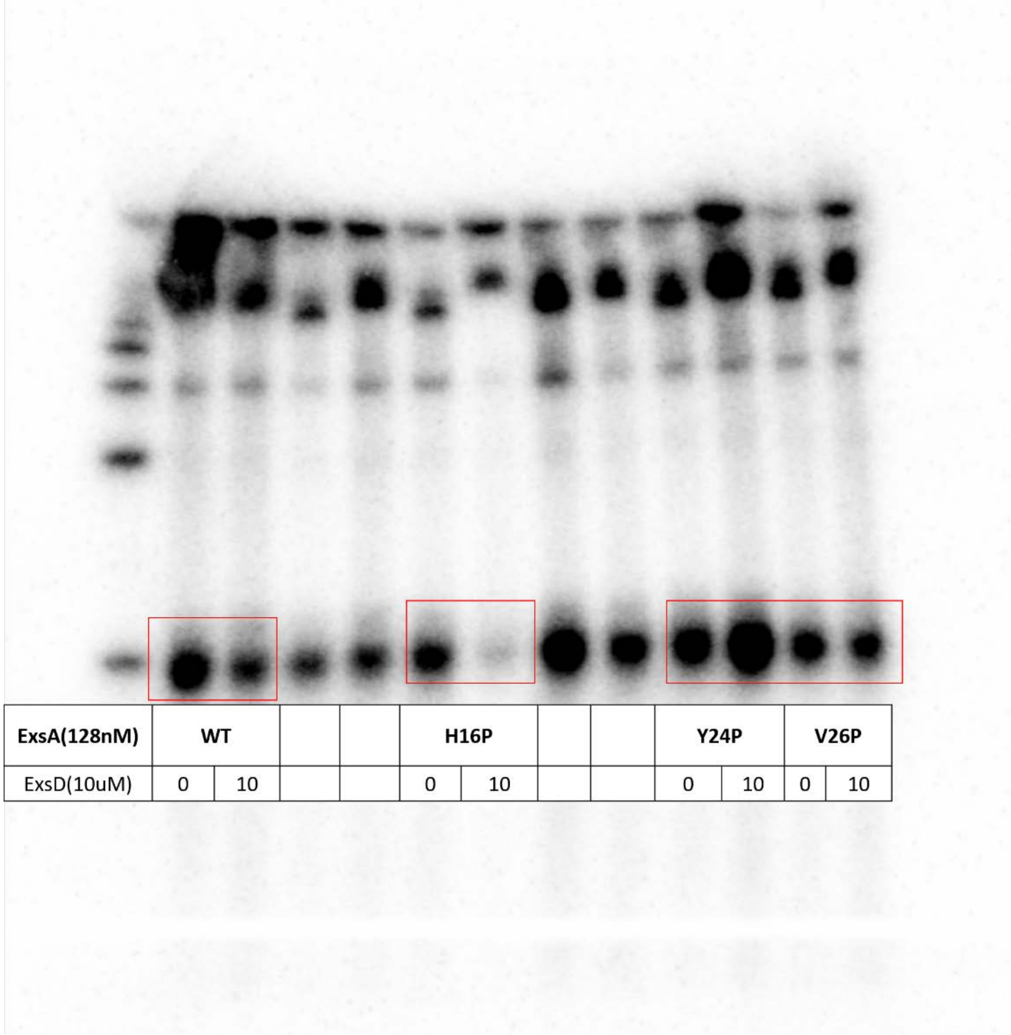

ExsA(128nM)	WT				H16P				Y24P		V26P	
ExsD(10uM)	0	10			0	10			0	10	0	10

S4.

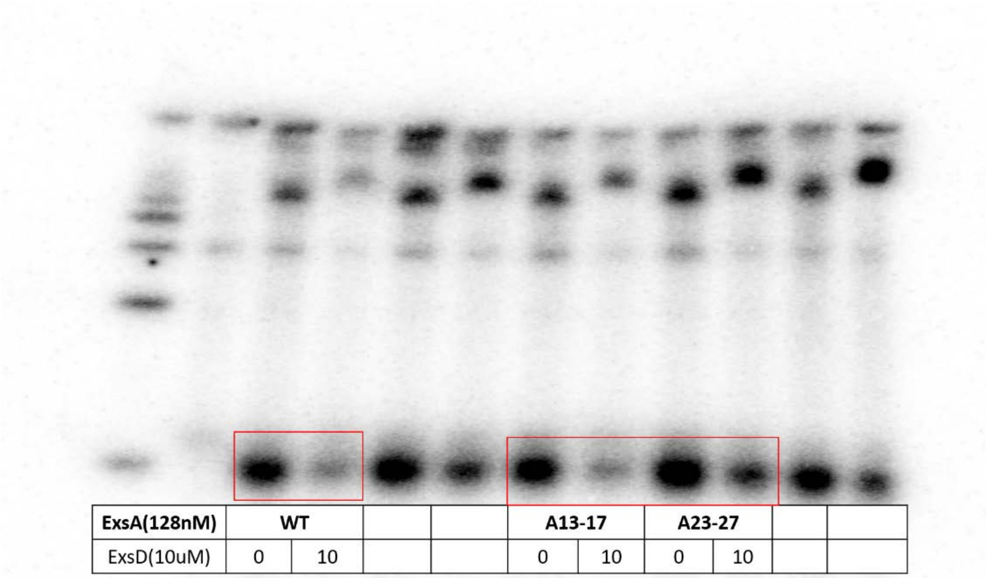

ExsA(128nM)	WT				A13-17		A23-27			
ExsD(10uM)	0	10			0	10	0	10		

S5.

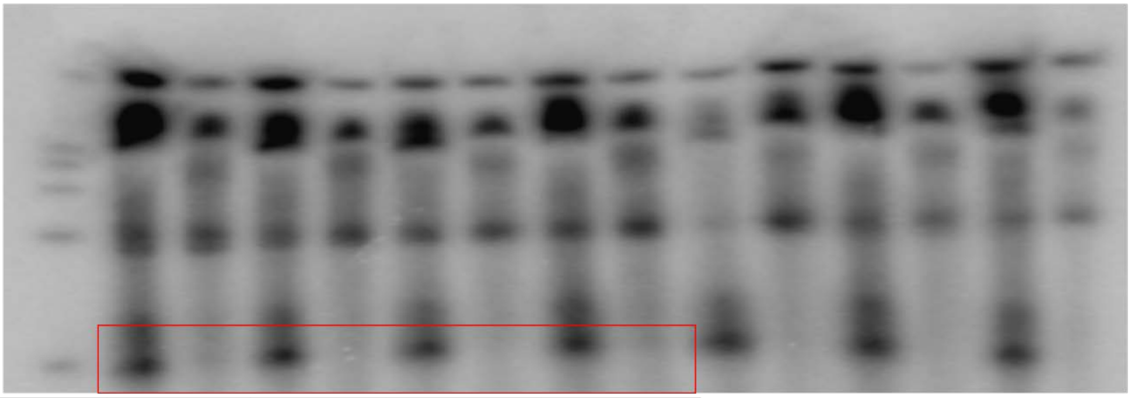

ExsA (128nM)	WT		T48A		L129A		L140A	
ExsD (10μM)	0	10	0	10	0	10	0	10

S6.

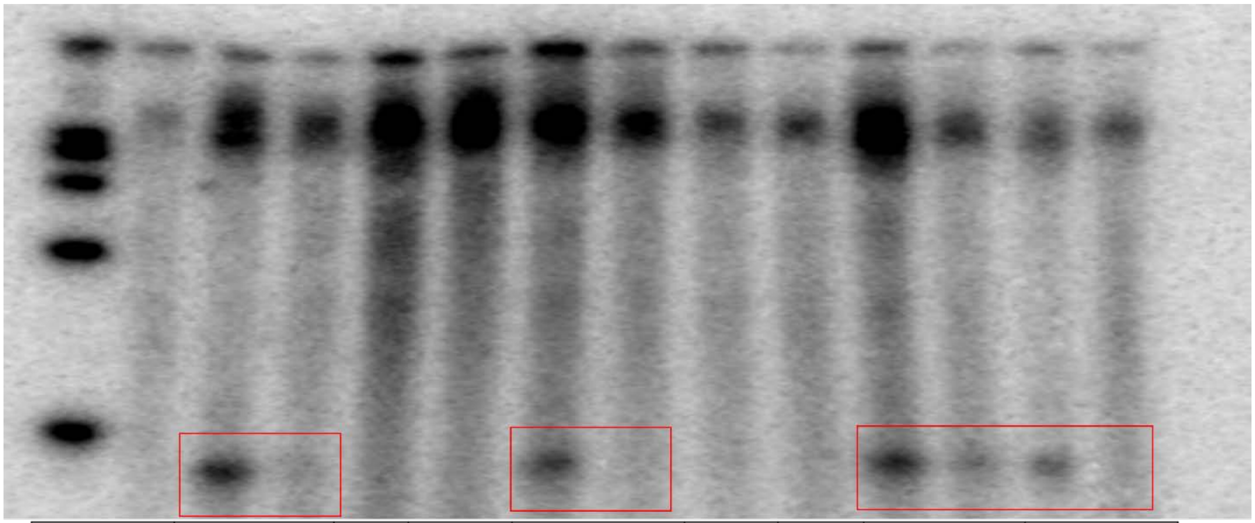

ExsA (128nM)	WT				Q90A				L95A		E98A	
ExsD (10µM)	0	10			0	10			0	10	0	10

S7.

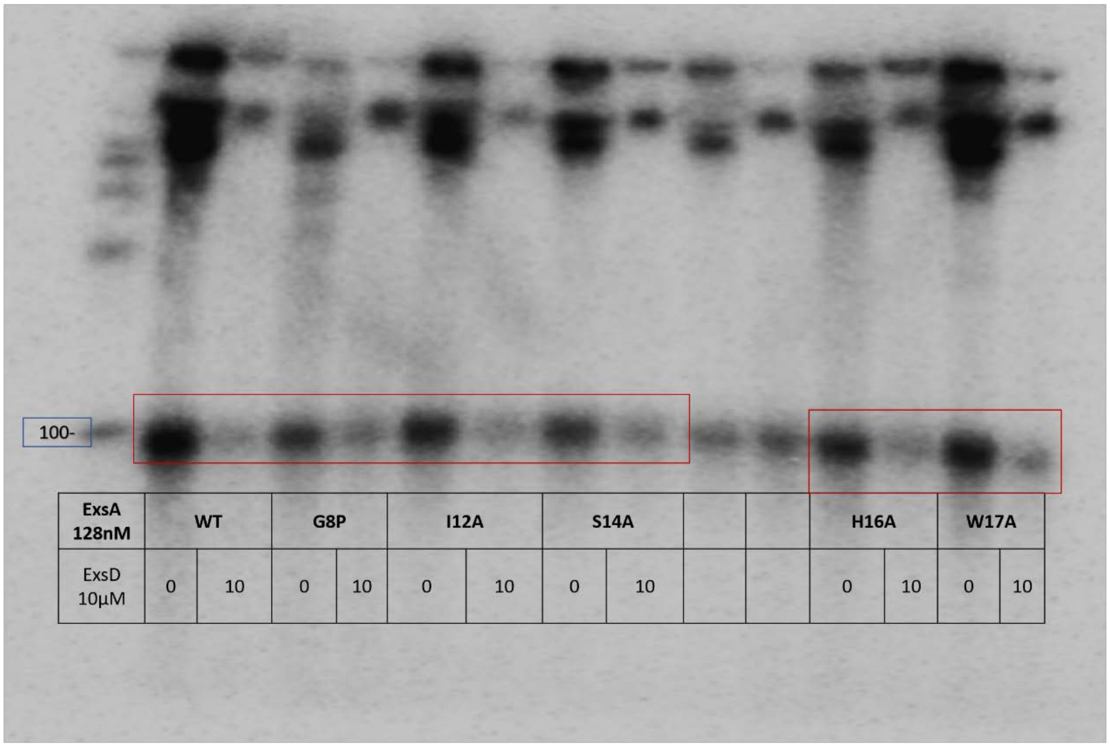

ExsA 128nM	WT		G8P		I12A		S14A				H16A		W17A	
ExsD 10µM	0	10	0	10	0	10	0	10			0	10	0	10

S8.

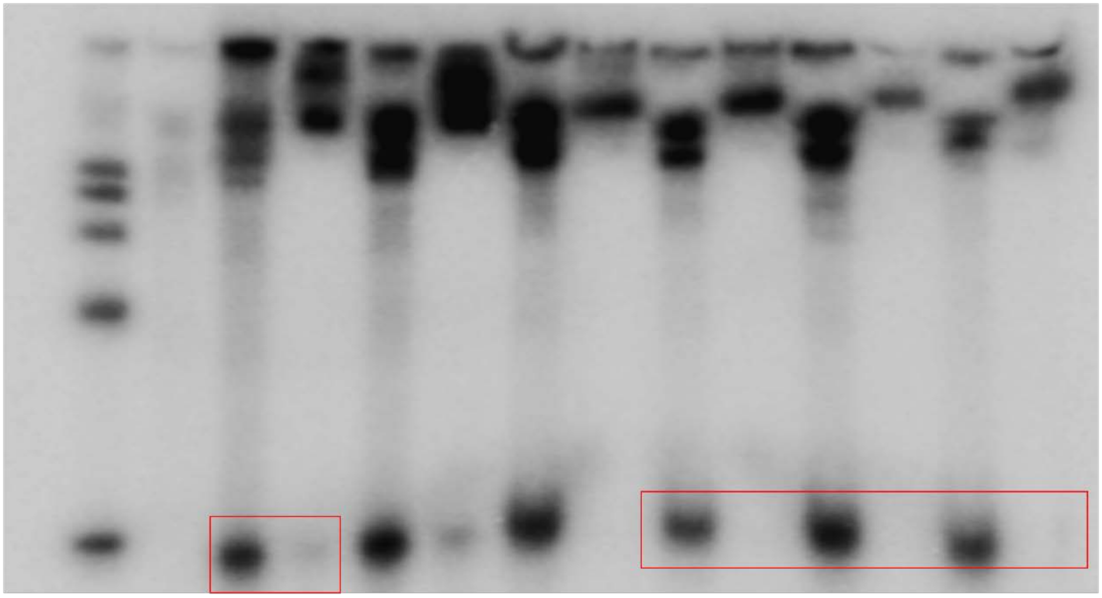

ExsA 128nM	WT						R9A		K10A		Q11A	
ExsD 10µM	0	10					0	10	0	10	0	10

S9.

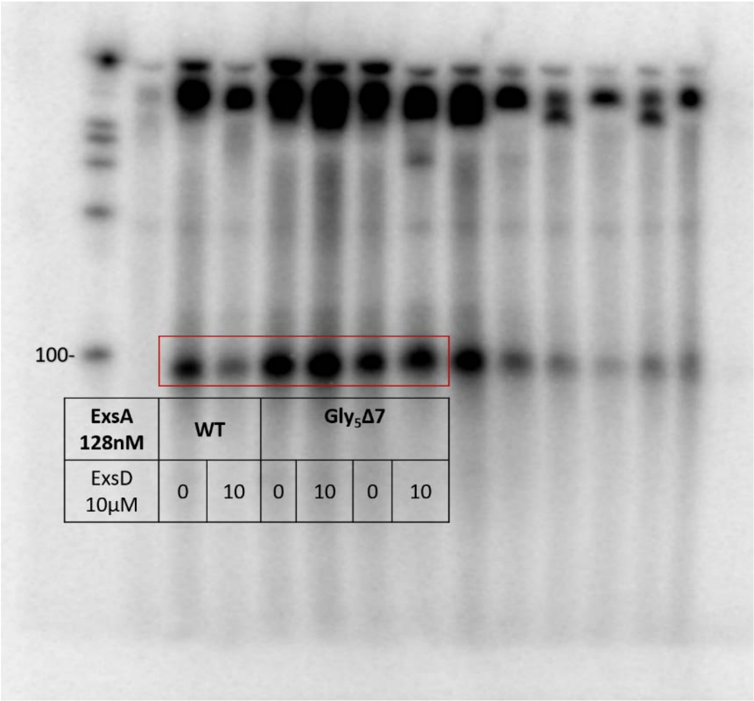

S10.

Images associated with Figure 3 in the Manuscript

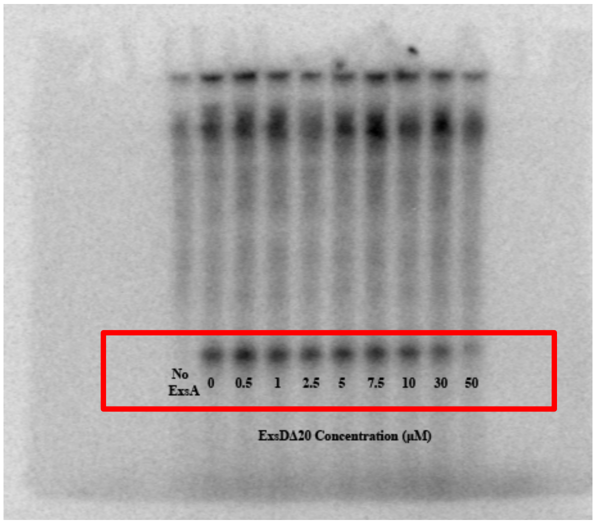

S11.

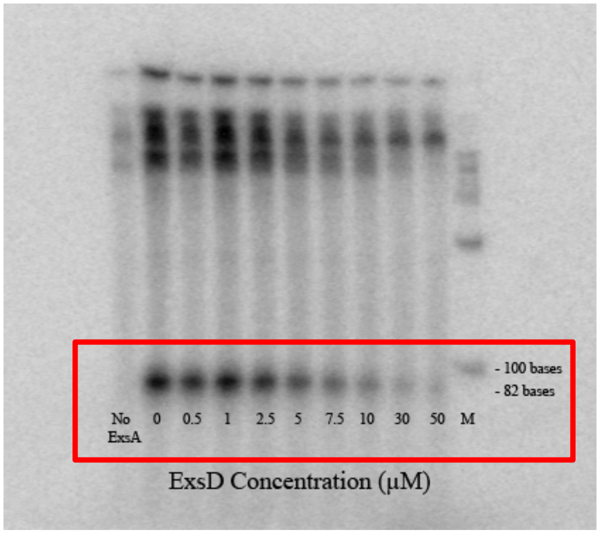

S12.

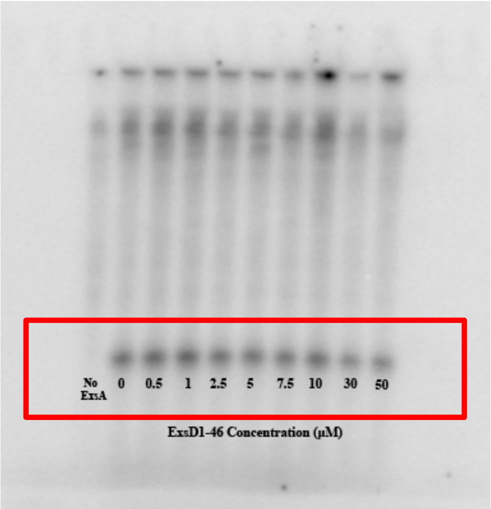

S13.

Uncropped image associated with S3 in this file

ExsA (nM)	-	128			
ExsD$_{1\text{-}38}$-MBP (μM)	-	0		10	20

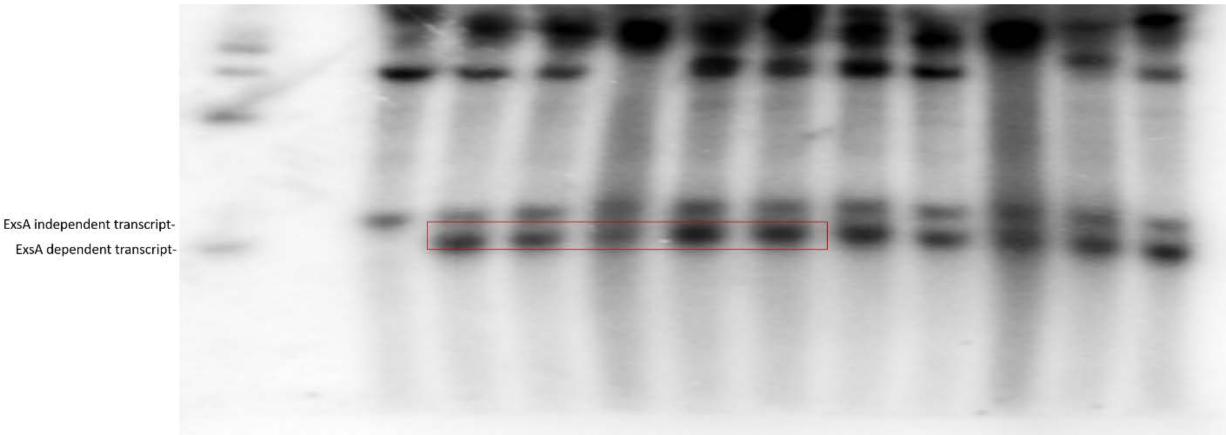

S14.

S15. The original uncropped SDS-PAGE Images of the coomassie stained gels shows in supplementary figure S2. are given below. Please note that the final molar concentrations of all ExsA and ExsD variants in the *in vitro* transcription assays were adjusted to be the same. The relevant bands are marked by red boxes and the lanes are labelled.

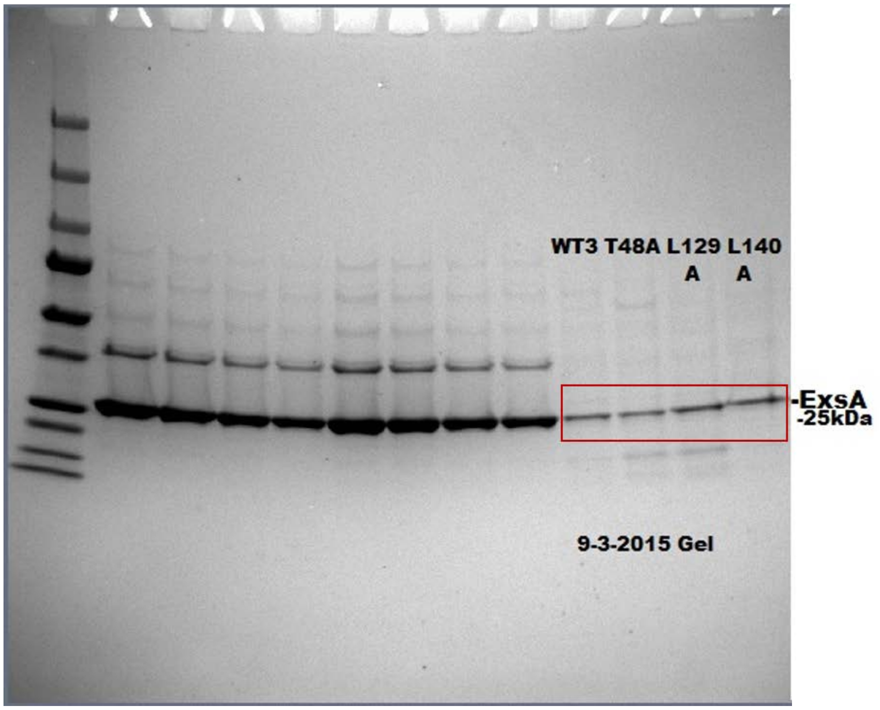

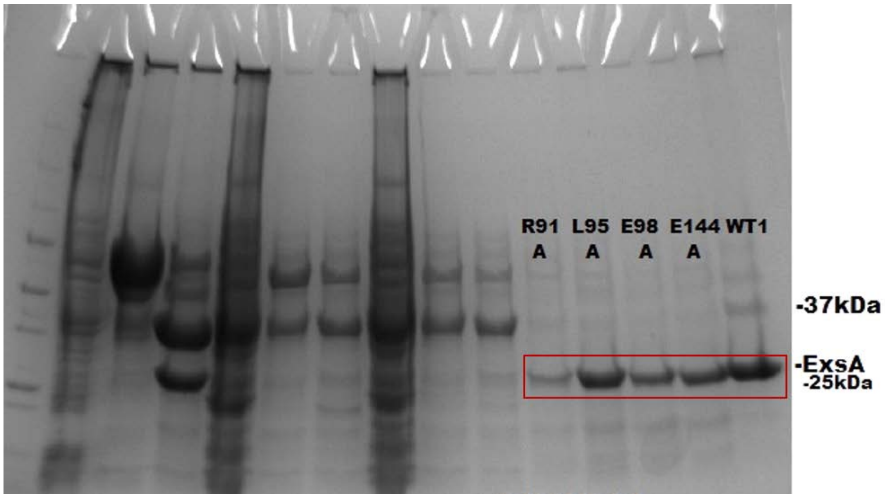

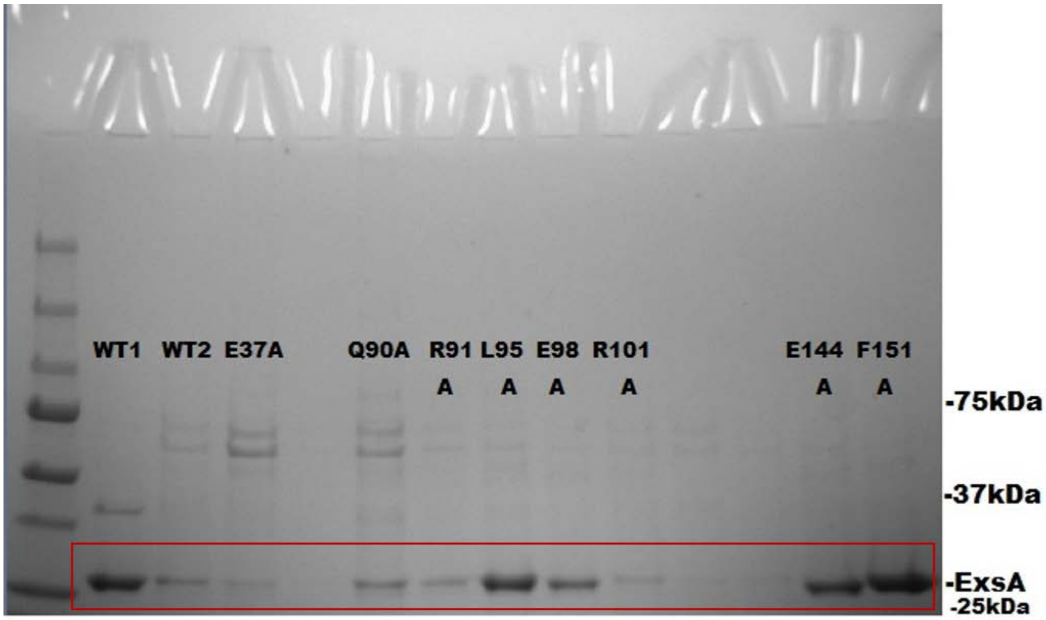
WT1
WT2
E37A
Q90A
R91 A
L95 A
E98 A
R101 A
E144 A
F151 A
-75kDa
-37kDa
-ExsA
-25kDa

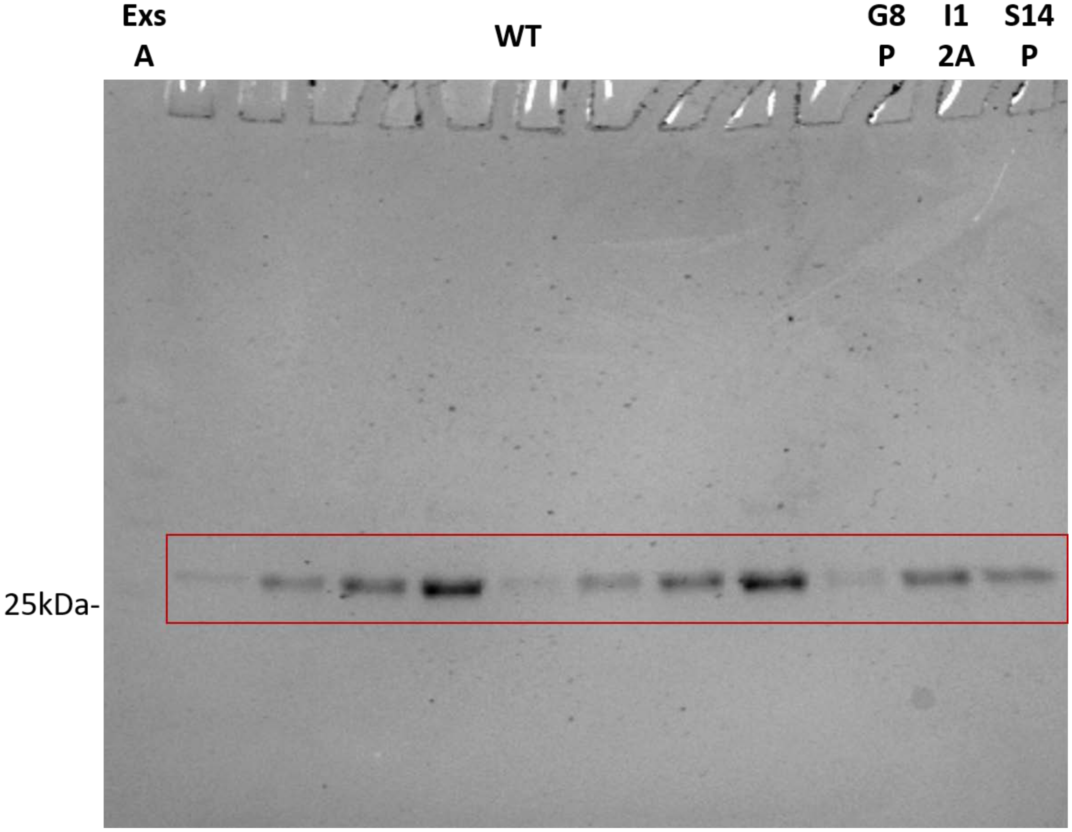
Exs A
WT
G8 P
I1 2A
S14 P
25kDa-

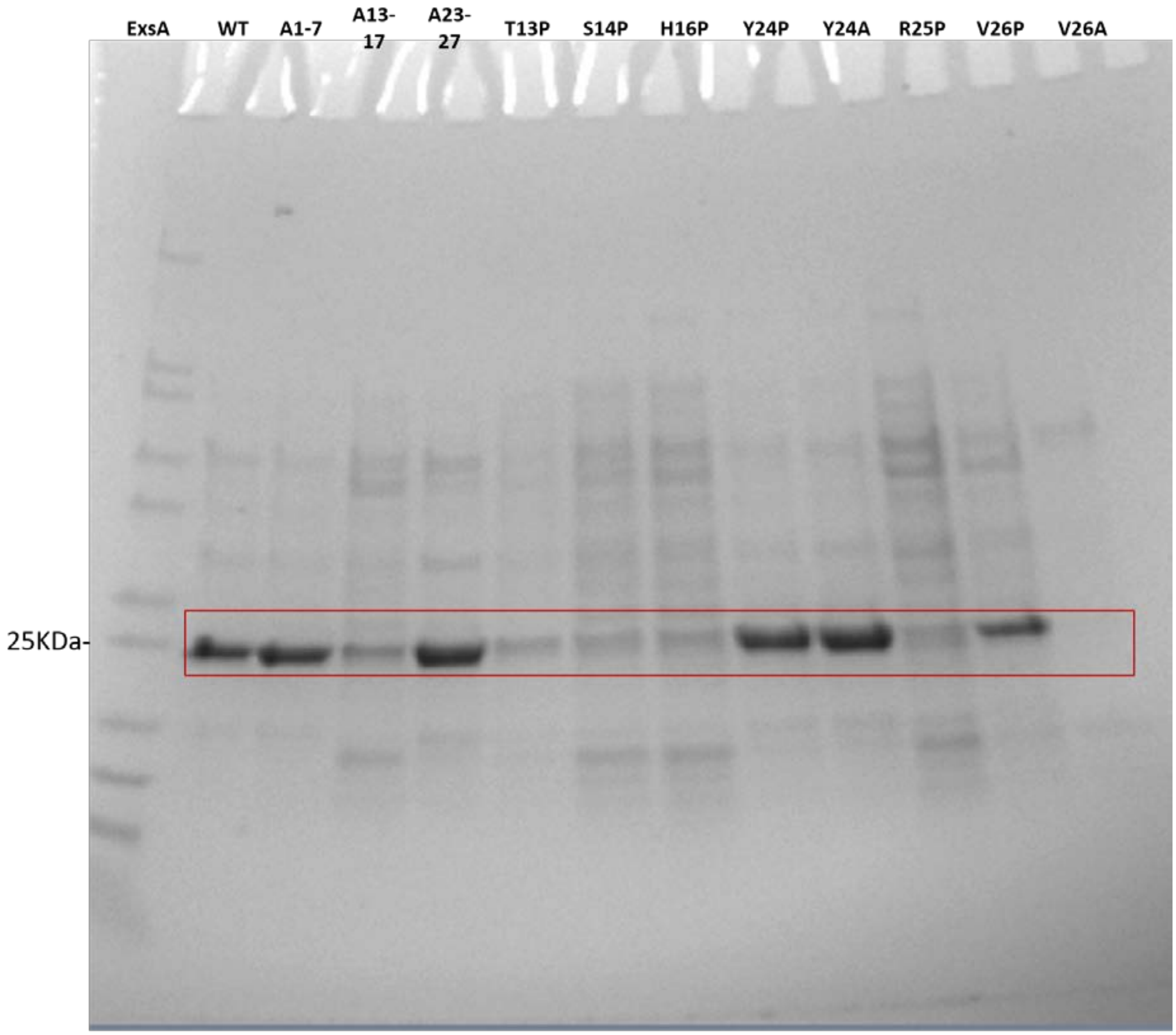
ExsA
WT
A1-7
A13-17
A23-27
T13P
S14P
H16P
Y24P
Y24A
R25P
V26P
V26A
25KDa-

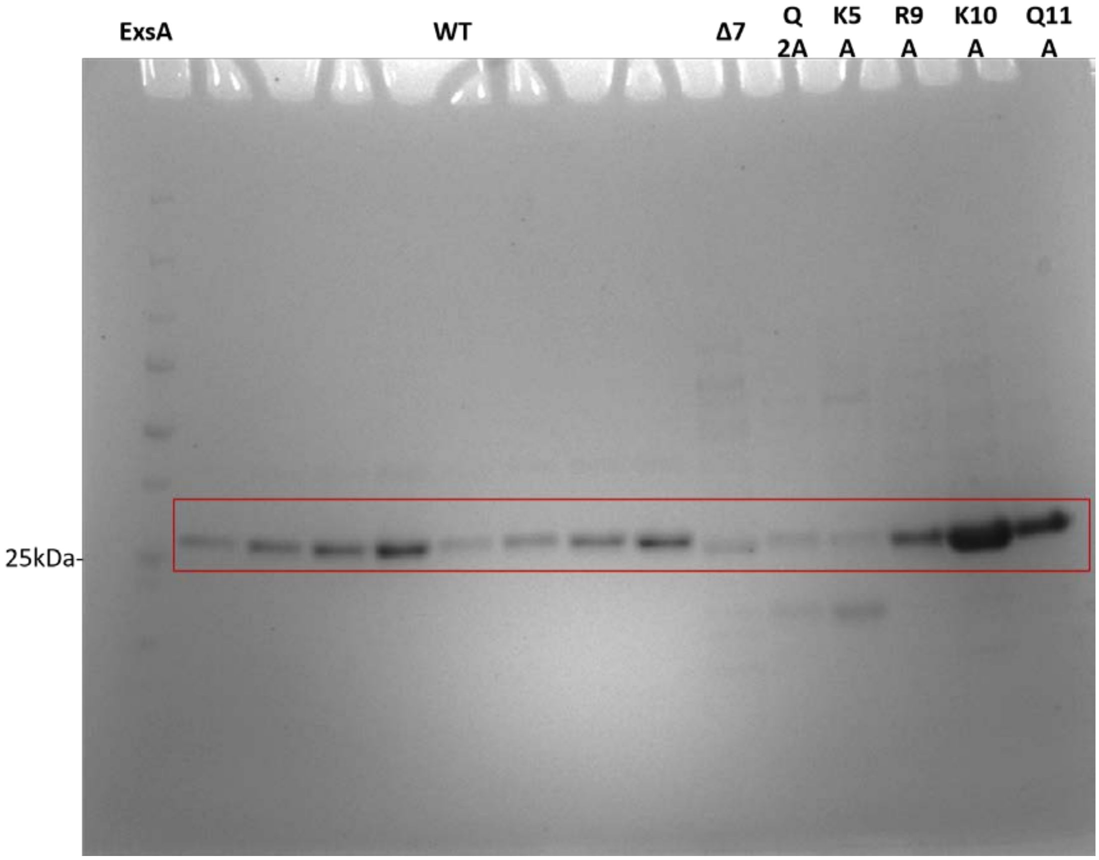
ExsA
WT
Δ7
Q 2A
K5 A
R9 A
K10 A
Q11 A
25kDa-

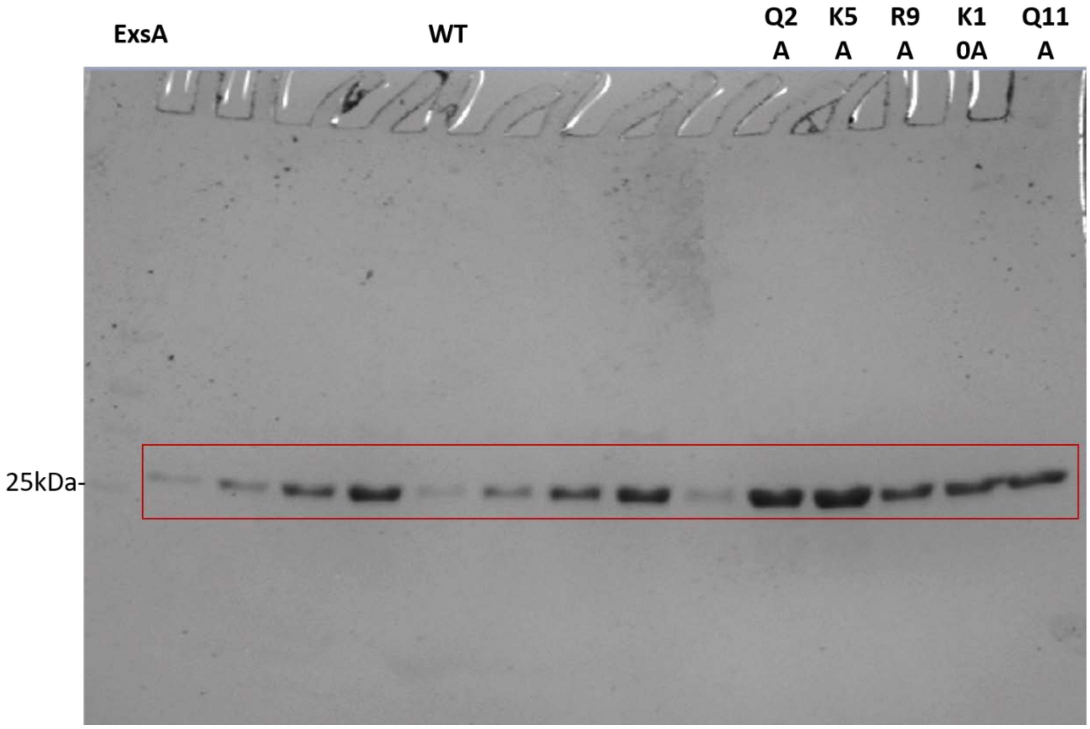
ExsA
WT
Q2 A
K5 A
R9 A
K1 0A
Q11 A
25kDa-

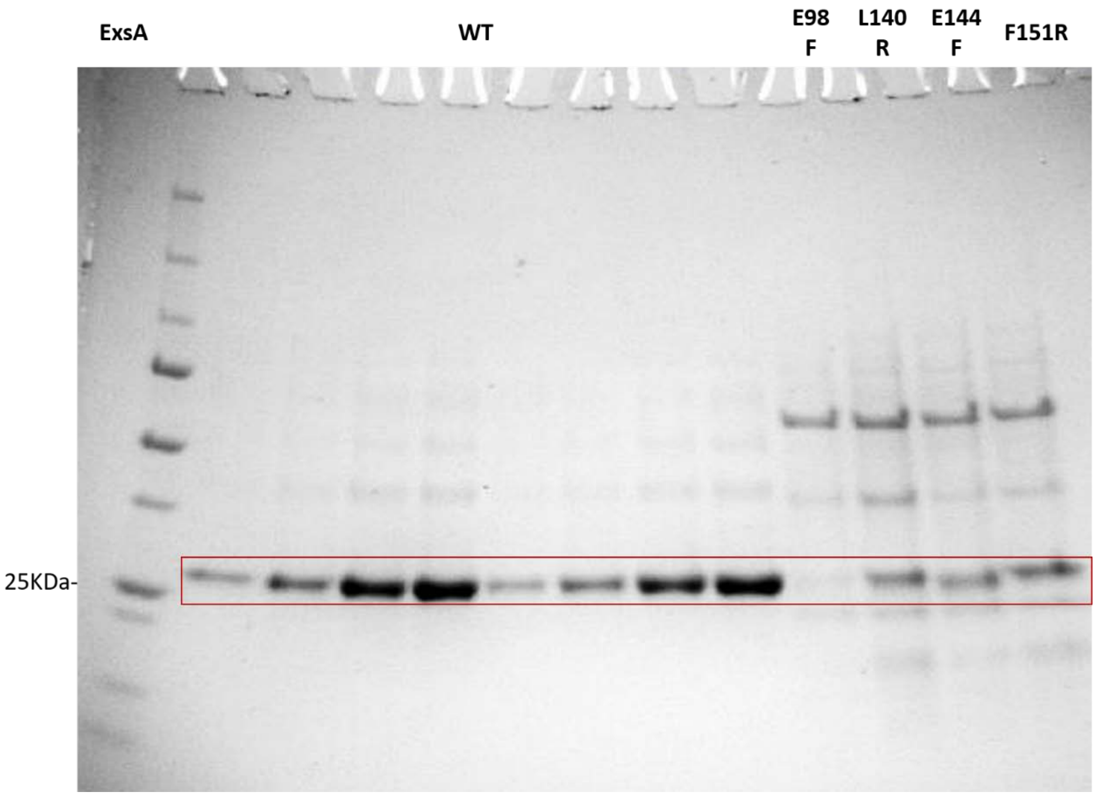
ExsA
WT
E98
F
L140
R
E144
F
F151R
25KDa-

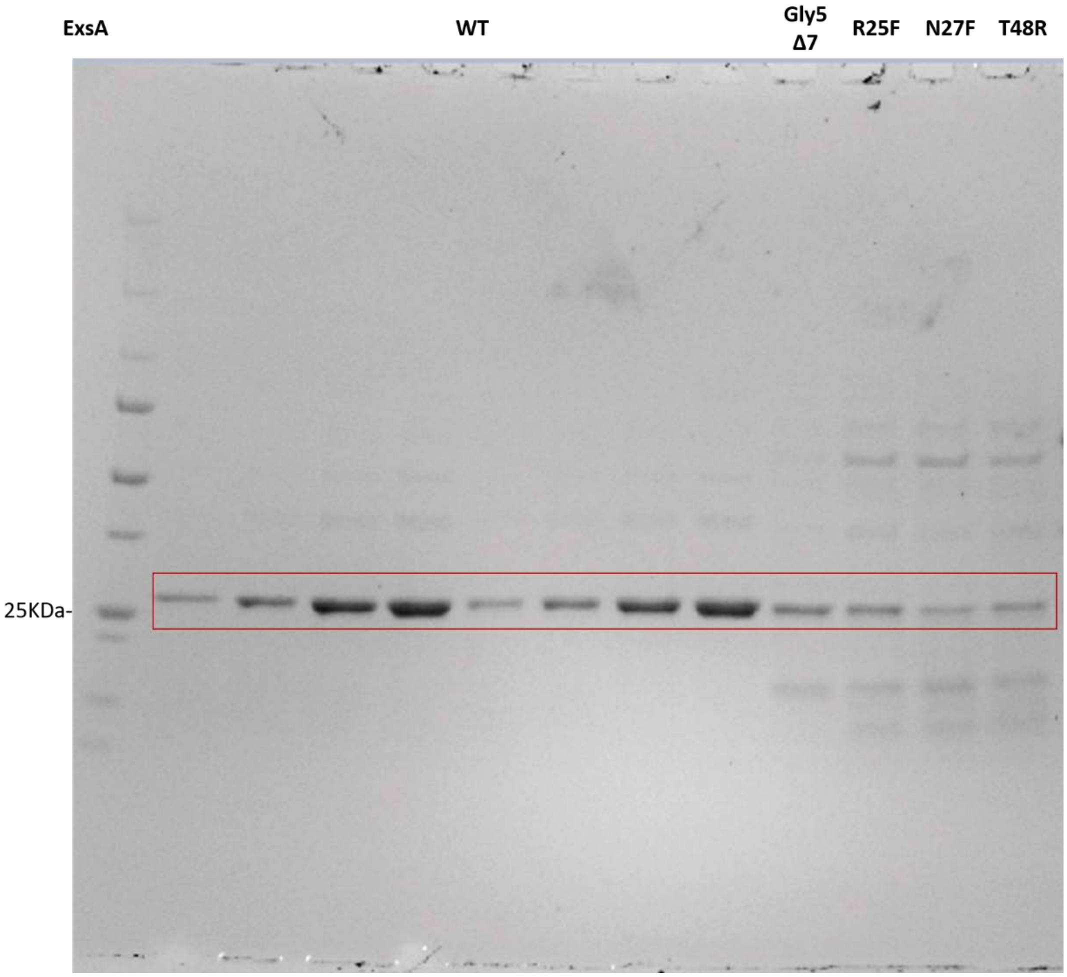
ExsA
WT
Gly5
Δ7
R25F
N27F
T48R
25KDa-

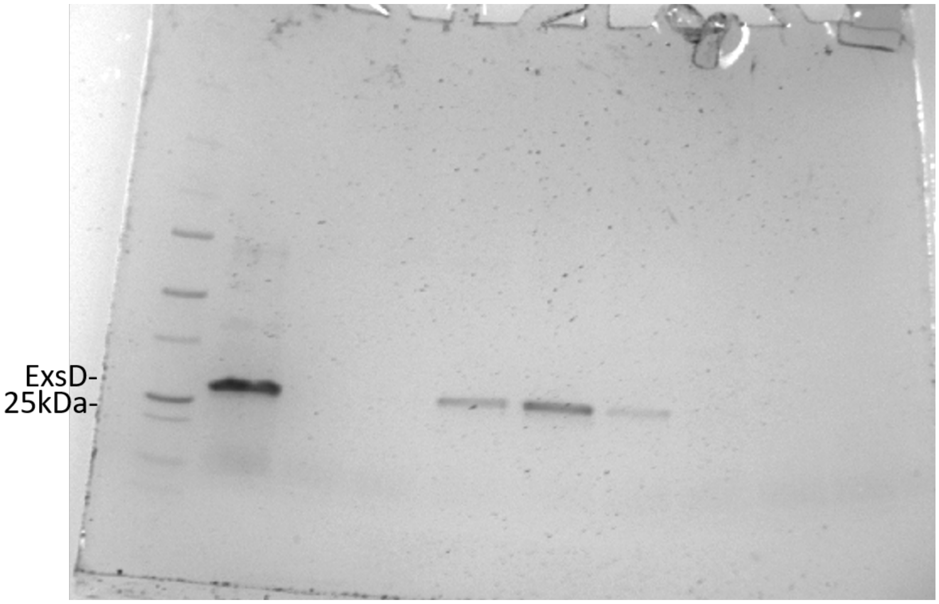
ExsD-
25kDa-

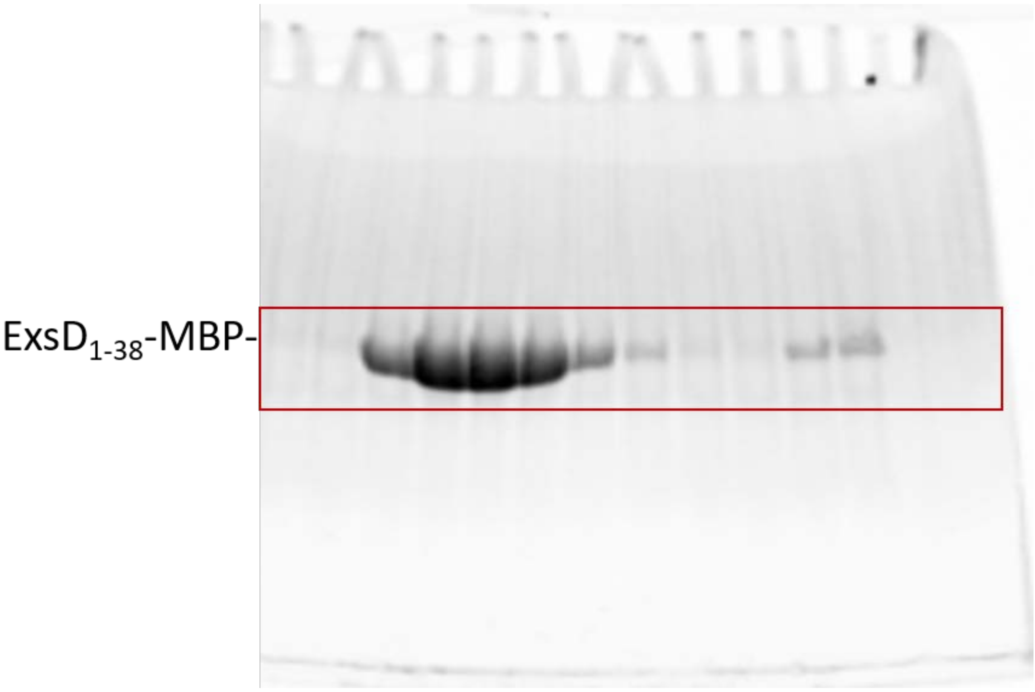
$ExsD_{1-38}$-MBP-
